# The growth factor FGF21 maintains neuromuscular junction through histone deacetylase HDAC4 in denervation-induced skeletal muscle atrophy

**DOI:** 10.1016/j.jbc.2025.110756

**Published:** 2025-09-23

**Authors:** Lirong Zheng, Takashi Sasaki, Liyang Ni, Tsutomu Hashidume, Mitsuki Kawabe, Yu Takahashi, Yoshio Yamauchi, Makoto Shimizu, Ryuichiro Sato

**Affiliations:** 1 Laboratory of Nutri-Life Science, Department of Applied Biological Chemistry, Graduate School of Agricultural and Life Sciences, The University of Tokyo, Tokyo, Japan; 2 Laboratory of Food Biochemistry, Department of Applied Biological Chemistry, Graduate School of Agricultural and Life Sciences, The University of Tokyo, Tokyo, Japan; 3 Laboratory of Nutrition and Life Science, Graduate School of Humanities and Sciences, Ochanomizu University, Tokyo, Japan; 4 Institute for Human Life Innovation, Ochanomizu University, Tokyo, Japan

**Keywords:** skeletal muscle atrophy, denervation, FGF21, neuromuscular junction, TGFB, fibro-adipogenic progenitors

## Abstract

Skeletal muscles undergo atrophy in response to denervation and neuromuscular disease. Understanding the mechanisms by which denervation drives muscle atrophy is crucial for developing therapies against neurogenic muscle atrophy. Here, we identified muscle-secreted fibroblast growth factor 21 (FGF21) as a key inducer of atrophy following muscle denervation. In denervated skeletal muscles, Fgf21 is the most robustly upregulated member of the Fgf family and acts in an autocrine/paracrine manner to promote muscle atrophy. Silencing Fgf21 in muscle prevents denervation-induced muscle wasting by preserving neuromuscular junction (NMJ) innervation. Conversely, forced expression of FGF21 in muscle reduces NMJ innervation, leading to muscle atrophy. Mechanistically, TGFB1 released by denervated fibro-adipogenic progenitors (FAPs) upregulates Fgf21 expression through the JNK/c-Jun axis. The resulting increase in FGF21 protein reduces the cytoplasmic level of histone deacetylase 4 (HDAC4), culminating in muscle atrophy. HDAC4 knockdown abolishes the atrophy-resistant effects observed in Fgf21-deficient denervated muscles, resulting in muscle atrophy. Our findings reveal a novel role and heretofore unrecognized mechanism of FGF21 in skeletal muscle atrophy, suggesting that inhibiting muscular FGF21 could be a promising strategy for mitigating skeletal muscle atrophy.

Skeletal muscle is a dynamic and metabolically active tissue that is essential for locomotion, posture, and systemic homeostasis. Proper innervation by motor neurons is critical for preserving muscle structure and function, and its disruption leads to neurogenic muscle atrophy. Neuromuscular diseases, such as amyotrophic lateral sclerosis (ALS), are characterized by progressive motor neuron degeneration, resulting in severe muscle wasting, functional decline, and ultimately death. Despite its clinical importance, there are currently no effective therapies to prevent or reverse muscle loss in neurogenic disorders. Thus, elucidating the molecular pathways that govern muscle mass maintenance is essential for developing targeted interventions against muscle-wasting diseases.

Skeletal muscle adapts to physiological and pathological cues through the secretion of myokines—cytokines that function in autocrine, paracrine, or endocrine manners (1). Among them, fibroblast growth factor 21 (FGF21), a member of the endocrine FGF subfamily along with FGF19 and FGF23, has emerged as a key metabolic regulator. Unlike canonical FGFs, FGF21 lacks a heparin-binding domain, enabling its systemic circulation (2). Although initially identified as a liver-derived hormone (3), FGF21 is also secreted by skeletal muscle (4). Extensive studies have demonstrated its beneficial effects in metabolic disorders, including obesity, type 2 diabetes, and non-alcoholic steatohepatitis (NASH), through actions that reduce fat mass and improve glucose and lipid metabolism (5, 6, 7). In skeletal muscle, FGF21 is secreted in response to various stressors, including exercise (8), starvation (9), autophagy impairment (10), mitochondrial dysfunction and aging (11). While several lines of evidence suggest that FGF21 may negatively impact muscle mass and function (9, 12), its role in neurogenic muscle atrophy remains poorly defined.

In this study, we identified a novel role for muscle-derived FGF21 in mediating denervation-induced muscle atrophy. We showed that FGF21 promotes neuromuscular junction (NMJ) disruption by reducing the cytoplasmic retention of HDAC4, thereby impairing NMJ maintenance and accelerating muscle wasting. Conversely, genetic ablation of Fgf21 enhances HDAC4 cytoplasmic localization, facilitates NMJ repair, and attenuates muscle loss. We further demonstrated that Fgf21 expression in denervated muscles is regulated by TGFB1/JNK/c-Jun signaling originating from fibro-adipogenic progenitors (FAPs). These findings establish FGF21 as a key mediator of neurogenic atrophy and a potential therapeutic target in neuromuscular diseases.

## Results

### FGF21 is upregulated in neurogenic atrophic skeletal muscles and is associated with muscle weakness

**Figure 1:**
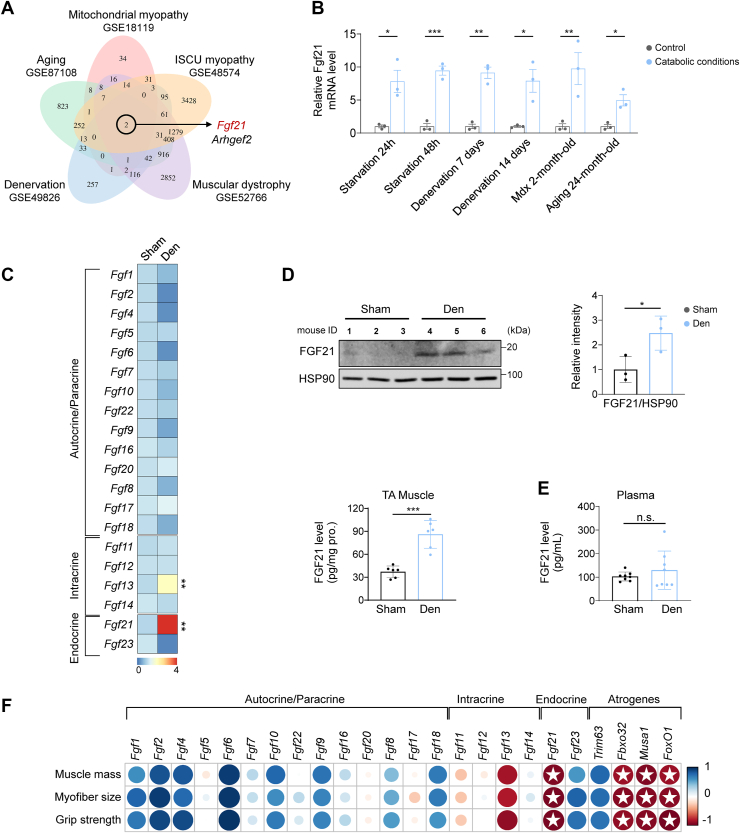
**FGF21 is upregulated in neurogenic atrophic skeletal muscles and is associated with muscle weakness.***A*, Venn diagram of overlapping upregulated genes in five muscle atrophic conditions. *B*, Fgf21 mRNA levels in the tibialis anterior (TA) muscle under starvation, denervation, dystrophy (mdx), and aging (n = 3). *C*, Heatmap of mRNA levels of Fgf members in TA muscle (n=3–5). *D*, protein levels of FGF21 in TA muscles detected in Western blot (n = 3) and ELISA (n = 6). *E*, ELISA analysis of FGF21 protein levels in plasma (n = 8). *F*, correlation analysis between skeletal muscle phenotypes and mRNA levels of Fgf family members and atrogenes (n = 5). Data were analyzed by two-tailed Student’s *t* test. ∗*p* < 0.05; ∗∗*p* < 0.01; ∗∗∗*p* < 0.001.

To identify regulators of neurogenic muscle atrophy, we re-analyzed five public datasets (GSE18119, GSE48574, GSE2766, GSE49826, and GSE87108), and identified consistent Fgf21 upregulation (Fig. 1*A*), which was also observed in our in-house models of starvation, denervation, dystrophy, and aging (Fig. 1*B*). Sciatic nerve transection in mice caused notable atrophy, as shown by reduced grip strength, muscle size, and fiber diameter, alongside elevated Fbxo32/Atrogin1 and Trim63/MuRF1 expression (Fig. S1, *A–F*). Among the FGF family members, only Fgf13 and Fgf21 were upregulated by denervation in tibialis anterior (TA) muscle, with Fgf21 showing the most pronounced increase (Fig. 1*C*). Immunoblot and ELISA confirmed elevated protein levels of FGF21 in denervated TA muscles (Fig. 1*D*). The expression of FGF21 receptors Klb, Fgfr1c, and Fgfr4 was also upregulated (Fig. S1*G*). Despite FGF21’s endocrine role, plasma FGF21 levels remained unchanged after denervation (Fig. 1*E*), and Fgf21 mRNA levels were specifically elevated in denervated muscles (Fig. S1*H*). Fgf21 induction was detectable by day 3 post-denervation and persisted for at least 32 days (Fig. S1*I*), with a similar response in denervated TA muscle of rats (Fig. S1*J*). Correlation analysis showed that Fgf21 expression was positively associated with atrogene levels and inversely correlated with muscle weight, size, and grip strength (Fig. 1*F*). Collectively, these findings suggest that muscular FGF21 may contribute to the progression of neurogenic muscle atrophy.

### Fgf21 deficiency protects skeletal muscles from denervation-induced atrophy

**Figure 2:**
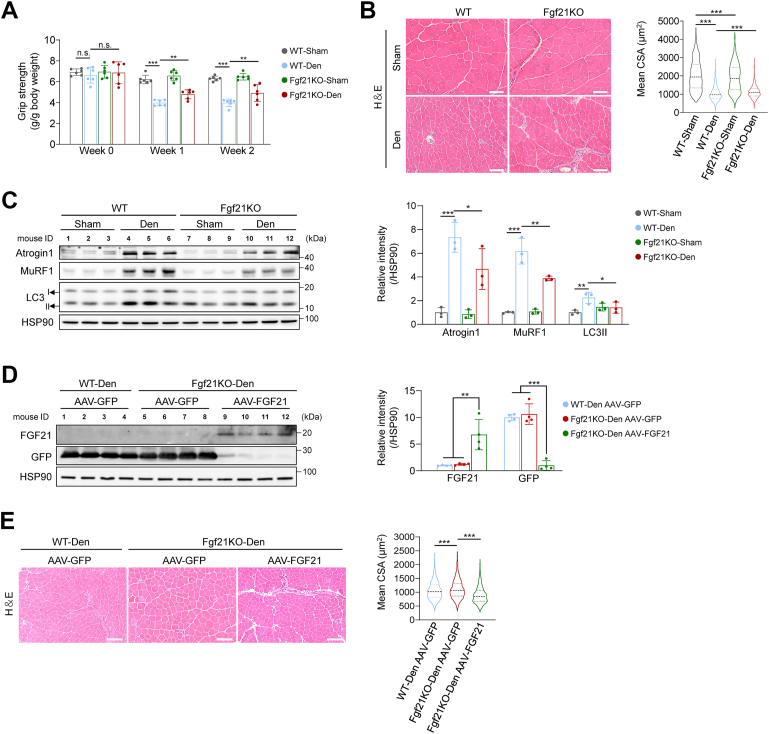
**Fgf21 deficiency protects skeletal muscles from denervation-induced atrophy.***A*, Four-limb grip strength (g/g body weight) of WT and Fgf21KO mice (n = 6). *B*, representative cross-sectional images of TA muscle from WT and Fgf21KO mice (n = 4). Scale bar, 50 μm. (*C*) Protein levels of protein degradation markers in TA muscle of WT and Fgf21KO mice (n = 3). *D*, protein levels of rescued FGF21 in TA muscle (n = 4). *E*, representative cross-sectional images of TA muscle after FGF21 rescue (n = 6). Scale bar, 50 μm. In (*A*-*C*), data were analyzed by two-way ANOVA followed by Sidak’s multiple comparisons test. No significant interaction between genotype and treatment was detected in (*A*) (week 0: F(1, 20) = 0.106, *p* = 0.748; week 1: F(1, 20) = 1.904, *p* = 0.183; week 2: F(1, 20) = 3.736, *p* = 0.068). A significant interaction between genotype and treatment was observed in (*B*) (F(1,5266) = 48.695, *p* < 0.0001). In (*C*), different interactions were detected (Atrogin1: F(1,8) = 3.990, *p* = 0.081; MuRF1: F(1,8) = 13.332, *p* = 0.006; LC3II: F(1,8) = 8.541, *p* = 0.019). In (*C*) and (*D*), data were analyzed by one-way ANOVA followed by Tukey’s multiple comparisons test. ∗*p* < 0.05; ∗∗*p* < 0.01; ∗∗∗*p* < 0.001.

To investigate the role of FGF21 in muscle atrophy, Fgf21 knockout (Fgf21KO) mice were subjected to denervation (Fig. S2*A*). Both basal and denervation-induced Fgf21 expression were abolished in Fgf21KO mice (Fig. S2*B*). Grip strength normalized by body weight showed no significant difference between WT and Fgf21KO mice before denervation (Fig. 2*A*). Following denervation, both genotypes exhibited a decline in grip strength, but Fgf21KO mice exhibited partial functional preservation (Fig. 2*A*). Relative muscle weight declined similarly in both genotypes (Fig. S2*C*). The mean cross-sectional area (CSA) of sham-operated TA muscle was smaller in Fgf21KO mice (Figs. 2*B* and S2*D*). However, Fgf21KO mice retained larger mean CSA and a greater proportion of large fibers than WT mice after denervation (Figs. 2*B* and S2*D*). Denervation-induced upregulation of protein degradation markers (Atrogin1, MuRF1, LC3II) was markedly attenuated in Fgf21KO muscle (Fig. 2*C*), suggesting that reduced proteolysis may underlie the preservation of muscle mass and strength.

Given the protective effect of Fgf21 deficiency against denervation-induced atrophy, we examined the consequences of Fgf21 overexpression. AAV-mediated FGF21 overexpression in TA muscle significantly elevated circulating FGF21 levels, regardless of denervation status (Fig. S3, *A*–*C*). FGF21 overexpression exacerbated muscle atrophy, as indicated by reduced grip strength, and smaller myofiber size, both at baseline and after denervation, whereas muscle weight was not further affected (Fig. S3, *D*–*F*).

To confirm the intramuscular role of FGF21, rescue experiments were conducted by overexpressing FGF21 in Fgf21-null TA muscles followed by denervation (Fig. 2*D*). While plasma FGF21 was undetectable in Fgf21KO mice, FGF21 overexpression restored its circulating levels (Fig. S3*G*). Although TA muscle weight was similar between GFP-transduced WT and Fgf21KO mice, FGF21 overexpression in Fgf21KO muscles reduced TA mass (Fig. S3*H*). Likewise, the enlarged CSA observed in denervated Fgf21KO muscles was abolished by FGF21 overexpression, shifting the fiber distribution toward smaller sizes (Figs. 2*E* and S3*I*). These data confirm that muscle-derived FGF21 promotes atrophy and that its absence confers resistance to denervation-induced muscle loss.

### Fgf21 deficiency improves NMJ innervation

**Figure 3:**
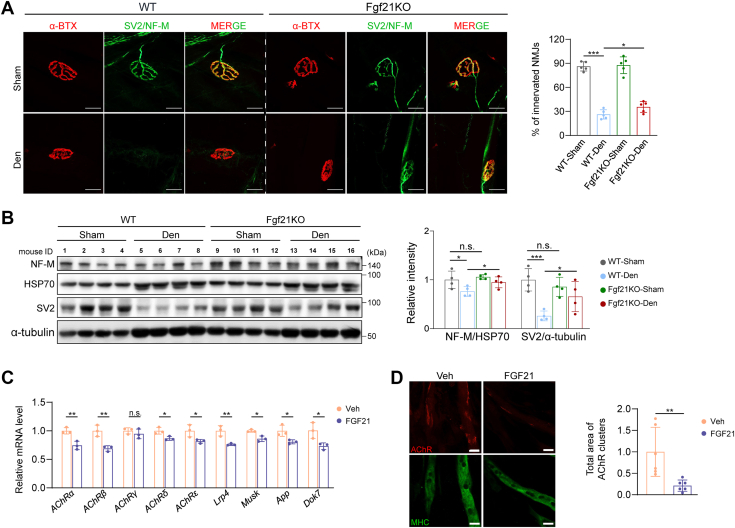
**Fgf21 deficiency improves NMJ innervation.***A*, representative images of neuromuscular junction (NMJ) innervation in the extensor digitorum longus (EDL) muscle of WT and Fgf21KO mice (n = 5). *B*, protein levels of NF-M and SV2 in EDL muscle of WT and Fgf21KO mice (n = 4). *C*, mRNA levels of NMJ-related genes in C2C12 myotubes treated with FGF21 (n = 3). *D*, representative images of AChR intensity in C2C12 myotubes (n = 6). Scale bar, 20 μm. In (*A*, *B*), data were analyzed by two-way ANOVA followed by Sidak’s multiple comparisons test. No significant interaction between genotype and treatment was detected in (*A*) (F(1, 16) = 1.654, *p* = 0.217). In (*B*), different interactions were detected (NF-M: F(1,12) = 0.797, *p* = 0.390; SV-2: F(1,12) = 5.918, *p* = 0.032). In (*C*, *D*), data were analyzed by two-tailed Student’s *t* test. ∗*p* < 0.05; ∗∗*p* < 0.01.

To understand how Fgf21 deficiency resists denervation-induced muscle atrophy, we performed RNA-Seq on TA muscles from WT and Fgf21KO mice 2 weeks post-denervation. Denervation and Fgf21 deletion led to distinct gene expression patterns (Fig. S4*A*), with overlapping and unique differentially expressed genes (DEGs) identified across four key comparisons: WT denervated vs. WT sham (WD vs. WS), Fgf21KO denervated vs. Fgf21KO sham (KD vs. KS), WT sham vs. Fgf21KO sham (WS vs. KS), and WT denervated vs. Fgf21KO denervated (WD vs. KD) (Fig. S4, *B* and *C*). KEGG pathway analysis revealed enrichment in Axon guidance, Neurotrophin signaling, Cytokine-receptor interactions, and Calcium signaling pathways (Fig. S4*D*), suggesting a role for FGF21 in neuromuscular signaling. Given the importance of axon guidance in neuromuscular junction (NMJ) maintenance, we assessed NMJ integrity. Denervation impaired NMJ innervation in WT mice, whereas Fgf21KO mice exhibited partial preservation (Fig. 3*A*). NMJ-associated proteins (NF-M and SV2) were reduced post-denervation in both genotypes but were better maintained in Fgf21KO mice (Fig. 3*B*). No differences were observed between genotypes under sham conditions (Fig. 3*B*). *In vitro*, recombinant FGF21 treatment suppressed the expression of NMJ-related genes and reduced postsynaptic AChR intensity along the myotubes (Fig. 3, *C* and *D*). These findings indicate that the muscle atrophy-resisting effect of Fgf21 deficiency is associated with improved NMJ innervation.

### Fgf21 deficiency resists muscle atrophy *via* HDAC4

**Figure 4:**
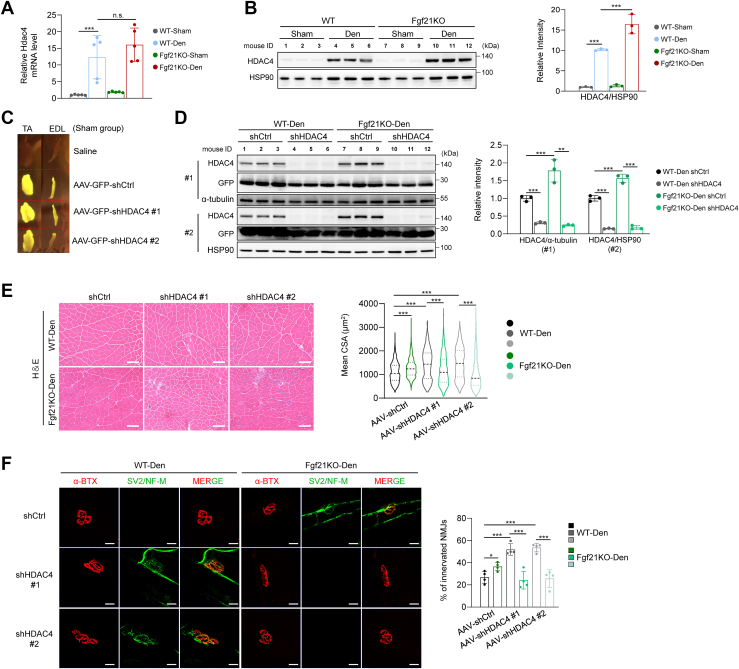
**Fgf21 deficiency resists muscle atrophy *via* HDAC4.***A and B*, mRNA (*A*) and protein (*B*) levels of Hdac4 in TA muscle of WT and Fgf21KO mice (n = 3–5). (*C*) Representative images of AAV-transduced GFP expression in TA and EDL muscles. *D*, protein levels of HDAC4 after knockdown in TA muscle of WT and Fgf21KO mice (n = 3). *E*, representative cross-sectional images of TA muscles from WT and Fgf21KO mice (n = 5). Scale bar, 50 μm. *F*, representative images of NMJ innervation in EDL muscle from WT and Fgf21KO mice (n = 4). Data were analyzed by two-way ANOVA followed by Sidak’s multiple comparisons test. No significant interaction between genotype and treatment was detected in (*A*) (F(1, 16) = 0.599, *p* = 0.450). A significant interaction between genotype and treatment was observed in (*B*) (F(1,8) = 18.359, *p* < 0.003). In (*D*-*F*), significant interactions between genotype and HDAC4 presence were detected (in (*D*), #1: F(1,8) = 19.462, *p* = 0.002; #2: F(1,8) = 37.962, *p* < 0.001; in (*E*), #1: F(1,4772) = 164.379, *p* < 0.001; #2: F(1,5662) = 450.588, *p* < 0.001; in (*F*), #1: F(1,12) = 41.400, *p* < 0.001; #2: F(1,12) = 46.698, *p* < 0.001). ∗*p* < 0.05; ∗∗*p* < 0.01; ∗∗∗*p* < 0.001.

Class IIa histone deacetylases (HDACs), particularly HDAC4, are critical regulators of neural activity and muscle responses to denervation. To elucidate the underlying mechanisms of enhanced NMJ maintenance, we examined the relationship between FGF21 and HDAC4. While Hdc4 mRNA levels increased with denervation and were unaffected by Fgf21 loss (Fig. 4*A*), HDAC4 protein levels were higher in Fgf21KO mice post-denervation (Fig. 4*B*). Other Class IIa HDACs were also upregulated by denervation but remained unaffected by Fgf21 deletion (Fig. S5*A*). To assess the functional role of HDAC4, AAV-shRNA targeting Hdac4 was delivered to TA muscles following denervation. Viral transduction was confirmed by GFP fluorescence (Fig. 4*C*), and efficient knockdown of HDAC4 was validated at the protein level (Figs. 4*D* and S5*B*). In non-denervated TA muscles, both Fgf21 deficiency and HDAC4 knockdown decreased fiber size, highlighting their roles under physiological conditions (Fig. S5*C*). Two weeks after denervation, control shRNA-injected WT muscles showed a 50% reduction in CSA compared with sham-operated muscles (Figs. 4*E* and S5*D*). Notably, HDAC4 knockdown in denervated WT muscles mitigated CSA loss, whereas in Fgf21KO mice, HDAC4 knockdown further exacerbated atrophy (Figs. 4*E* and S5*D*), suggesting a context-dependent function of HDAC4. NMJ maintenance was enhanced by either Fgf21 deficiency or HDAC4 knockdown in denervated WT muscles but was abrogated when Hdac4 was silenced in Fgf21-null muscles (Fig. 4*F*). Neither Fgf21 deficiency nor HDAC4 knockdown affected NMJ morphology under sham conditions (Fig. S5*E*).

**Figure 5:**
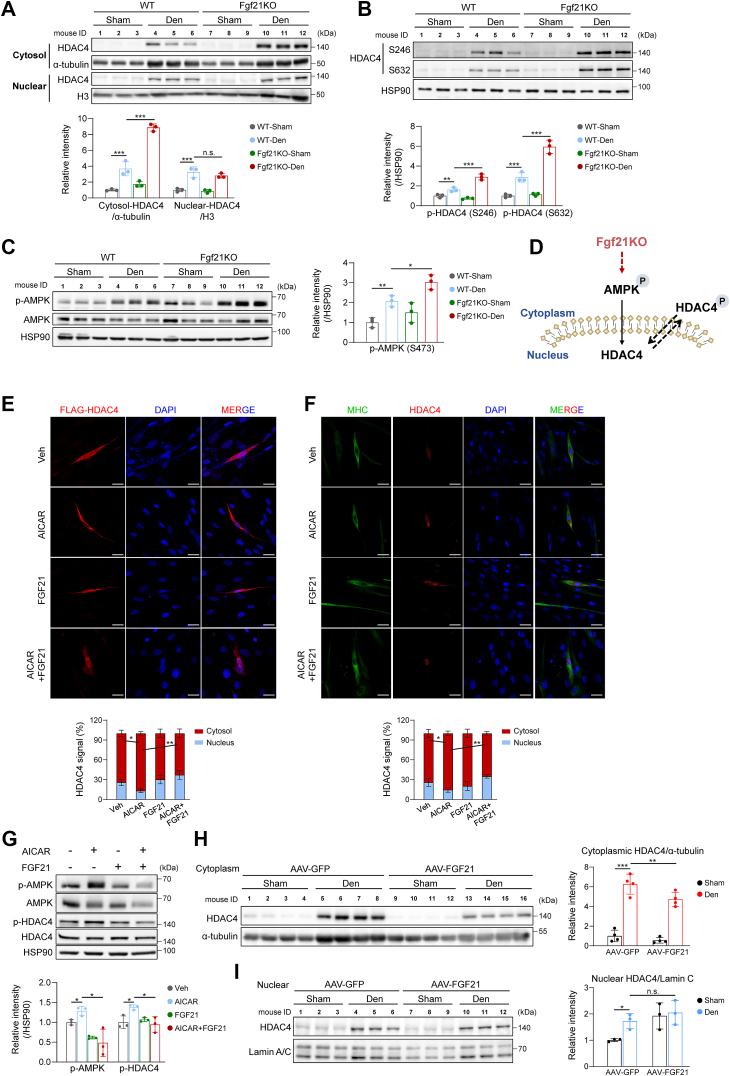
**FGF21 regulates HDAC4 localization *via* AMPK.***A*, protein levels of subcellular HDAC4 in TA muscle of WT and Fgf21KO mice (n = 3). *B* and *C*, protein levels of HDAC4 and AMPK in TA muscle of WT and Fgf21KO mice (n = 3). *D*, proposed regulatory model of FGF21 on HDAC4. *E* and *F*, C2C12 cells were pretreated with 0.5 mM AICAR for 1 h followed by the addition of 1 μg/ml of recombinant human FGF21 protein for 6 h. Representative images of FLAG-HDAC4 and endogenous HDAC4 localization (*red*). Nuclei are shown in *blue* (DAPI) (n = 3). Scale bars, 20 μm. *G*, C2C12 cells were pretreated with 0.5 mM AICAR for 30 min followed by FGF21 protein treatment for 30 min. Protein levels of AMPK and HDAC4 in C2C12 myotubes treated with FGF21 and AICAR (n = 3). *H* and *I*, protein levels of subcellular HDAC4 in TA muscles injected with AAV-GFP or AAV-FGF21 (n = 3 or 4). In (*A*-*C*) and (*H*,*I*), data were analyzed by two-way ANOVA followed by Sidak’s multiple comparisons test. In (*A*-*C*), different interactions between genotype and treatment were detected (in (*A*), Cytosol HDAC4: F(1,8) = 52.009, *p* < 0.001; Nuclear HDAC4: F(1,8) = 0.424, *p* = 0.533; in (*B*), HDAC4 S246: F(1,8) = 41.806, *p* < 0.001; HDAC4 S632: F(1,8) = 34.802, *p* < 0.001; in (*C*), F(1,8) = 1.204, *p* = 0.304). In (*E*-*G*), data were analyzed by one-way ANOVA followed by Tukey’s multiple comparisons test. No significant interactions between genotype and virus treatment were detected in (*H*, *I*) (Cytosol HDAC4: F(1,12) = 2.467, *p* = 0.142; Nuclear HDAC4: F(1,8) = 1.997, *p* = 0.195). ∗*p* < 0.05; ∗∗*p* < 0.01; ∗∗∗*p* < 0.001.

We then investigated the post-translational regulation of HDAC4, focusing on its phosphorylation-dependent subcellular localization. Denervation increased both cytoplasmic and nuclear HDAC4 levels in WT muscles, whereas Fgf21 deficiency further elevated cytoplasmic HDAC4 levels without affecting nuclear levels (Fig. 5*A*). Corresponding increases in HDAC4 phosphorylation at the two serine sites were observed (Fig. 5*B*). AMPK, a known HDAC4 kinase, showed increased phosphorylation following denervation and was further activated in Fgf21KO muscles (Fig. 5*C*), suggesting AMPK-mediated HDAC4 regulation upon Fgf21 deletion (Fig. 5*D*). In C2C12 cells, AMPK activation by AICAR (5-Aminoimidazole-4-carboxamide ribonucleoside) promoted cytoplasmic HDAC4 localization, which was reversed by FGF21 addition, shifting HDAC4 to the nucleus (Fig. 5, *E* and *F*). FGF21 also suppressed AICAR-induced phosphorylation of AMPK and HDAC4 (Fig. 5*G*). *In vivo*, AAV-mediated FGF21 overexpression reduced cytoplasmic HDAC4 levels without altering nuclear levels (Fig. 5, *H* and *I*), confirming the regulation of FGF21 on AMPK-mediated HDAC4 localization. These data suggest that AMPK-mediated cytoplasmic retention of HDAC4 contributes to the muscle-sparing effects in Fgf21-deficient muscles.

### TGFB1 promotes myotube atrophy *via* FGF21-mediated NMJ damage

**Figure 6:**
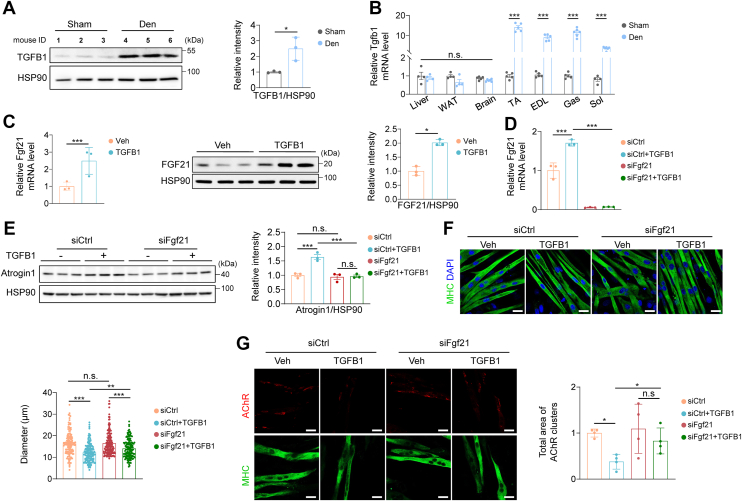
**TGFB1 promotes myotube atrophy *via* FGF21-mediated NMJ damage.***A*, protein levels of TGFB1 in TA muscle (n = 3). *B*, mRNA levels of Tgfb1 in different tissues (n = 4–6). *C*, mRNA and protein levels of Fgf21 in C2C12 myotubes treated with TGFB1 (n = 3). *D*, mRNA levels of Fgf21 in C2C12 myotubes treated with siFgf21 and TGFB1 (n = 3). *E*, protein levels of Atrogin1 in C2C12 myotubes treated with siFgf21 and TGFB1 (n = 3). *F*, representative images of C2C12 myotubes treated with siFgf21 and TGFB1 (n = 3). Scale bar, 20 μm. *G*, representative images of AChR intensity treated with siFgf21 and TGFB1 (n = 4). Scale bar, 20 μm. In (*A*-*C*), data were analyzed by two-tailed Student’s *t* test. In (*D*-*G*), data were analyzed by two-way ANOVA followed by Sidak’s multiple comparisons test. Significant interactions between Fgf21 presence and compound treatment were detected (in (*D*), F(1,8) = 32.097, *p* < 0.001; in (*E*), F(1,8) = 19.036, *p* = 0.002; in (*F*), F(1,621) = 4.500, *p* = 0.034; in (*G*): F(1,12) = 1.551, *p* = 0.237). ∗*p* < 0.05; ∗∗*p* < 0.01; ∗∗∗*p* < 0.001.

To explore how Fgf21 is regulated during denervation-induced muscle atrophy, RNA-seq analysis of denervated TA muscles revealed enrichment of TGFB and MAPK pathways (Fig. S4*D*), implicating TGFB signaling in Fgf21 induction. Denervation significantly increased TGFB1 protein levels in TA muscle (Figs. 6*A* and S6*A*) and upregulated Tgfb1 mRNA levels specifically in skeletal muscles (Fig. 6*B*), without affecting plasma TGFB1 levels (Fig. S6*A*). In C2C12 myotubes, TGFB1 treatment elevated Fgf21 expression at both mRNA and protein levels (Fig. 6*C*), as well as its secretion (Fig. S6*B*). TGFB1 induced muscle atrophy, as evidenced by decreased myotube diameter, reduced MHC protein level, and increased Atrogin1 and MuRF1 expression (Fig. S6, *C* and *D*). FGF21 treatment also promoted muscle atrophy in C2C12 myotubes (Fig. S6, *E* and *F*), suggesting that TGFB1 drives FGF21-dependent muscle atrophy.

To test our hypothesis, Fgf21 was silenced in C2C12 cells before TGFB1 treatment. Knockdown of Fgf21 suppressed both basal and TGFB1-induced Fgf21 expression (Fig. 6*D*), attenuated Atrogin1 induction, and restored myotube diameter (Fig. 6, *E* and *F*). Notably, Fgf21 silencing had no effect under basal conditions, indicating that its role is specific to stress-induced atrophy (Fig. 6, *E* and *F*). Furthermore, TGFB1-induced Fgf21 expression occurred only in differentiated myotubes, but not in myoblasts (Fig. S6*G*), suggesting fiber-specific regulation.

We also investigated whether TGFB1 induces NMJ damage through FGF21. TGFB1 treatment reduced NMJ-related gene expression and AChR intensity (Fig. S6, *H* and *I*), whereas Fgf21 silencing partially rescued AChR loss (Fig. 6*G*). These findings identify FGF21 as a key downstream effector of TGFB1 in promoting both muscle fiber atrophy and NMJ deterioration.

### TGFB1 regulates Fgf21 expression through non-canonical JNK/c-Jun axis

**Figure 7:**
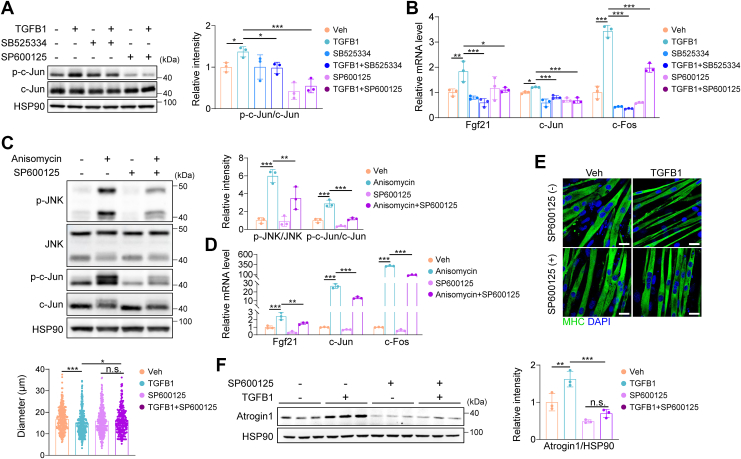
**TGFB1 regulates Fgf21 expression through non-canonical JNK/c-Jun axis.***A and B*, C2C12 cells were pretreated with 5 μM SB525334 or 10 μM SP600125 for 1 h, followed by treatment with 5 ng/ml TGFB1 for either 1 h (for Western blot) or 4 h (for qPCR). Protein levels of c-Jun and the mRNA levels of Fgf21, c-Jun, and c-Fos in C2C12 myotubes are shown (n = 3). *C and D*, C2C12 cells were pretreated with 10 μM SP600125 for 1 h followed by 5 μM Anisomycin treatment for either 30 min (for Western blot) or 1 h (for qPCR). Protein levels of JNK and c-Jun and mRNA levels of Fgf21, c-Jun, and c-Fos in C2C12 myotubes are shown (n = 3). (*E*,*F*) C2C12 cells were pretreated with 500 nM SP600125 for 1 h, followed by 5 ng/ml TGFB1 for 24 h. *E*, representative images of C2C12 myotubes treated with SP600125 and TGFB1 (n = 3). Scale bar, 20 μm. *F*, protein levels of Atrogin1 in C2C12 myotubes treated with SP600125 and TGFB1 (n = 3). Data were analyzed by two-way ANOVA followed by Sidak’s multiple comparisons test. Different interactions between compound and inhibitors treatment were detected (in (*A*), F(1,12) = 1.786, *p* = 0.209; in (*B*), Fgf21: F(1,12) = 6.576, *p* = 0.012; c-Jun: F(1,12) = 3.414, *p* = 0.067; c-Fos: F(1,12) = 95.109, *p* < 0.001; in (*C*), JNK ratio: F(1,8) = 6.756, *p* = 0.032; c-Jun ratio: F(1,8) = 15.172, *p* = 0.005; in (*D*), Fgf21: F(1,8) = 0.635, *p* = 0.449; c-Jun: F(1,8) = 63.009, *p* < 0.001; c-Fos: F(1,8) = 386.663, *p* < 0.001; in (*E*), F(1,1235) = 8.668, *p* = 0.003; in (*F*), F(1,8) = 4.429, *p* = 0.068). ∗*p* < 0.05; ∗∗*p* < 0.01; ∗∗∗*p* < 0.001.

To elucidate how TGFB1 induces Fgf21 expression during muscle atrophy, we examined the canonical and non-canonical TGFB signaling pathways (Fig. S7*A*). In C2C12 myotubes, TGFB1 elevated the expression of Smad target genes, which were suppressed by TGFB type I receptor inhibitor SB525334 and partially by Smad3 inhibitor SIS3 or by Smad3 knockdown (Fig. S7, *B*–*D*). However, Fgf21 induction was only partially inhibited by SB525334 and was unaffected by SIS3 or Smad3 knockdown (Fig. S7, *B*–*D*), suggesting Smad-independent regulation of Fgf21 expression. We then investigated the non-canonical pathways (Fig. S7, *E*–*H*). TGFB1 enhanced JNK activation, as shown by increased c-Jun phosphorylation, which was blocked by both SB525334 and JNK inhibitor SP600125 (Fig. 7*A*). These inhibitors also suppressed TGFB1-induced c-Jun, c-Fos, and Fgf21 expression (Fig. 7*B*). Activating JNK with Anisomycin increased JNK/c-Jun phosphorylation and Fgf21 expression, which were reversed by SP600125 (Fig. 7, *C* and *D*). *In vivo*, denervation elevated JNK and c-Jun protein levels in TA muscle (Fig. S7*I*). Moreover, JNK inhibition attenuated TGFB1-induced Atrogin1 expression and restored myotube diameter (Fig. 7, *E* and *F*). These findings demonstrate that TGFB1 regulates Fgf21 expression through the non-canonical JNK/c-Jun axis, rather than the canonical Smad pathway, to promote muscle atrophy.

### Denervation-activated FAPs secrete TGFB1 to promote muscle atrophy *via* FGF21

**Figure 8:**
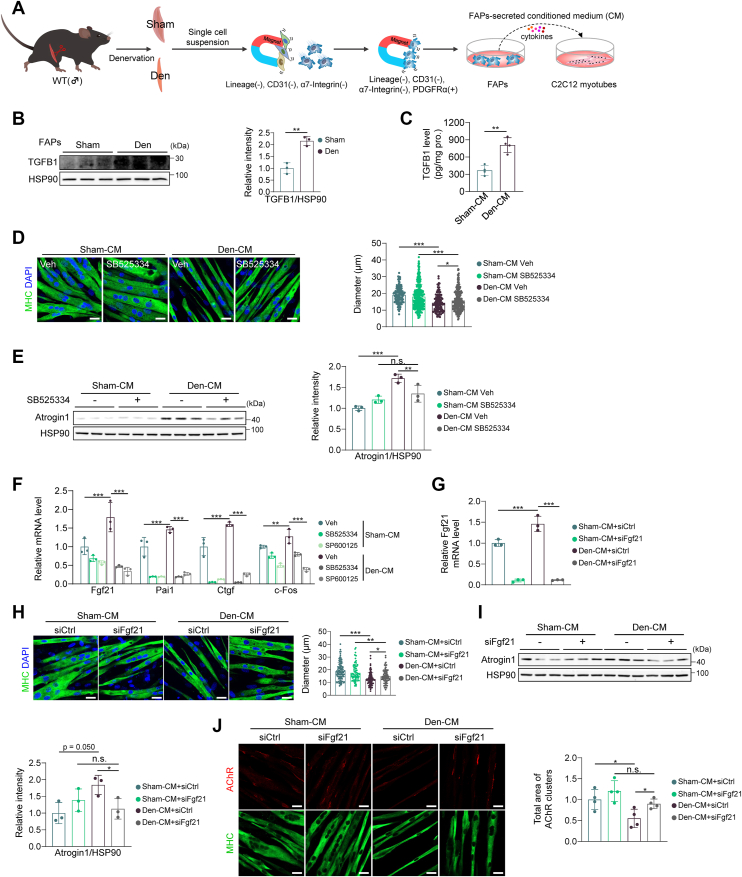
**Denervation-activated FAPs secrete TGFB1 to promote muscle atrophy *via* FGF21.***A*, schematic diagram of FAPs isolation and conditioned medium (CM) collection. *B*, protein levels of TGFB1 in FAPs (n = 3). *C*, protein levels of TGFB1 in CM were detected by ELISA (n = 4). *D and E*, C2C12 cells were pretreated with 5 μM SB525334 for 1 h followed by conditioned medium (CM) treatment for 24 h. *D*, representative images of C2C12 myotubes treated with CM and SB525334 (n = 3). Scale bar, 20 μm. *E*, protein levels of Atrogin1 in C2C12 myotubes treated with CM and TGFB1 (n = 3). (*F*) C2C12 cells were pretreated with 5 μM SB525334 or 10 μM SP600125 for 1 h followed by CM treatment for 4 h mRNA levels of Fgf21, Pai1, c-Jun, and c-Fos in C2C12 myotubes are shown (n = 3). *G*, mRNA levels of Fgf21 in C2C12 myotubes treated with CM and siFgf21 (n = 3). *H*, representative images of C2C12 myotubes treated with CM and siFgf21 (n = 3). Scale bar, 20 μm. *I*, protein levels of Atrogin1 in C2C12 myotubes treated with CM and siFgf21 (n = 3). *J*, representative images of AChR intensity in C2C12 myotubes treated with CM and siFgf21 (n = 4). Scale bar, 20 μm. In (*B*, *C*), data were analyzed by two-tailed Student’s *t* test. In (*D*-*J*), data were analyzed by two-way ANOVA followed by Sidak’s multiple comparisons test. In (*D*-*F*), different interactions between conditioned medium and inhibitor treatment were detected (in (*D*), F(1,813) = 6.915, *p* = 0.009; in (*E*), F(1,8) = 15.919, *p* = 0.004; in (*F*), Fgf21: F(1,12) = 13.840, *p* < 0.001; Pai1: F(1,12) = 7.787, *p* = 0.007; Ctgf: F(1,12) = 14.062, *p* < 0.001; c-Fos: F(1,12) = 6.495, *p* = 0.012). In (*G*-*J*), different interactions between conditioned medium and Fgf21 presence were detected (in (*G*), F(1,8) = 14.386, *p* = 0.005; in (*H*), F(1,387) = 7.911, *p* = 0.005; in (*I*), F(1,8) = 5.657, *p* = 0.045; in (*J*), F(1,12) = 0.459, *p* = 0.511). ∗*p* < 0.05; ∗∗*p* < 0.01; ∗∗∗*p* < 0.001.

To identify the source of denervation-upregulated TGFB1, we focused on fibro-adipogenic progenitors (FAPs), which are known to secrete Activin A (a member belonging to the TGFB superfamily) to promote muscle atrophy. FAPs were isolated from denervated (Den-FAPs) and sham-operated muscles (Sham-FAPs) using MACS (Figs. 8*A* and S8*A*). The number of FAPs was increased in denervated muscles compared to sham muscles (Fig. S8*B*). Den-FAPs exhibited significantly higher Tgfb1 expression at both mRNA and protein levels (Figs. 8*B* and S8*C*), and Den-FAPs-derived conditioned medium (Den-CM) contained higher TGFB1 levels than Sham-CM (Fig. 8*C*). Treatment of C2C12 myotubes with Den-CM reduced myotube diameter and increased Atrogin1 levels (Fig. S8, *D* and *E*), the effects that were reversed by TGFB signaling inhibitor SB525334 (Fig. 8, *D* and *E*). These data indicate that TGFB1 released from FAPs contributes to muscle atrophy.

Treatment of C2C12 myotubes with Den-CM increased the expression of TGFB target genes and Fgf21, all of which were suppressed by SB525334 and SP600125 (Fig. 8*F*). Silencing Fgf21 mitigated Den-CM-induced myotube atrophy and reduced Atrogin1 levels (Fig. 8, *G*–*I*). Den-CM treatment also reduced AChR intensity in C2C12 myotubes compared to that in Sham-CM group (Fig. S8*F*). Silencing Fgf21 restored AChR intensity (Fig. 8*J*), indicating that TGFB1 released by Den-FAPs promotes muscle atrophy through FGF21-mediated NMJ damage. These data demonstrate that FAPs are activated upon denervation to release TGFB1, which induces FGF21 expression in myofibers, leading to muscle atrophy.

## Discussion

Since its discovery in 2000 (13), FGF21 has been recognized as a stress-induced hormone and a therapeutic target for obesity and related metabolic syndromes (5, 6, 7). Notably, muscle-targeted AAV1-FGF21 gene therapy reverses metabolic dysfunction-associated steatohepatitis (MASH) progression in mice (14), but its role in muscle remains incompletely understood.

While FGF21 promotes myogenesis (15), mitochondrial function (16), and protects against inflammation-mediated muscle atrophy in murine myotubes (17), other studies have reported its induction of atrophy *via* mitophagy or HPA axis activation (9, 12), highlighting that the effects of FGF21 on skeletal muscle are highly context-dependent and may vary across physiological and pathological conditions.

In this study, we identified FGF21 as a key mediator of neurogenic atrophy. We found that Fgf21 expression is inversely correlated with muscle mass and function following denervation. Genetic ablation of Fgf21 significantly alleviates muscle atrophy following denervation by promoting cytoplasmic accumulation of HDAC4, which enhances NMJ maintenance, thereby contributing to muscle homeostasis. Mechanistically, Fgf21 induction in response to denervation is driven by TGFB1-mediated JNK/c-Jun signaling, originating from muscle-resident FAPs. The autocrine/paracrine action of FGF21 within skeletal muscle, as observed in our model, likely reflects the local metabolic adaptation to sciatic nerve transection, which predominantly affects innervated limb muscles. Given that skeletal muscle is rich in structural proteins and glycogen, FGF21-induced autophagy may help mobilize internal energy stores to sustain muscle viability during atrophy, without requiring systemic metabolic input.

The NMJ, a specialized synapse that transmits action potentials from motor neurons to muscle fibers, comprises three key components: the pre-synaptic motor nerve terminal, synaptic basal lamina, and post-synaptic muscle membrane (18). While FGF21 has been implicated in central nervous system functions such as memory regulation (19), and dietary preference (20), its role in the peripheral nervous system remains controversial. Some studies suggest that FGF21 promotes peripheral nerve regeneration following injury (21), whereas others report that it inhibits myelin development in Schwann cells (22).

In our study, pharmacological administration of recombinant FGF21 suppressed AChR expression in muscle cells, contributing to NMJ disruption and muscle atrophy. These findings suggest that FGF21 may exert divergent effects depending on the cellular differentiation state, with distinct functions in proliferative *versus* terminally differentiated muscle cells—a hypothesis that warrants further investigation. Notably, the protective effect of Fgf21 ablation on muscle wasting appears to be mediated by AMPK. Our observation that AMPK activation improves NMJ integrity is consistent with recent reports implicating AMPK as a central mediator of motoneuron and NMJ maintenance. Genetic, pharmacological, or physiological activation of AMPK has been shown to promote beneficial remodeling in various neuromuscular disorders (NMDs) (23).

The FGF21/AMPK axis mediates its effects partly by stimulating HDAC4 phosphorylation, a Class IIa histone deacetylase, resulting in its cytoplasmic retention. While HDAC4 regulates muscle development, differentiation, and fiber-specific transcriptional programs (24), the functional implications of its subcellular localization in skeletal muscle atrophy remain controversial, likely due to differences in the atrophic context, as distinct upstream signals and effector pathways may be involved in different muscle atrophy models. Nuclear HDAC4 represses MEF2-dependent transcription (25), whereas cytoplasmic HDAC4 may influence muscle homeostasis through non-transcriptional mechanisms (26, 27). Based on the current literature, we speculate that HDAC4 function in muscle atrophy may depend on the subcellular localization of its targets, thereby affecting muscle homeostasis in a localization-dependent manner. For example, Luo *et al.* (27). identified cytoplasmic substrates of HDAC4, including MyHC (myosin heavy chain) and Hsc70 (heat shock cognate 71 kDa protein). HDAC4 deacetylates MyHC isoforms, which are the primary components of the thick filaments in the contractile sarcomeres. MyHC isoforms are constitutively acetylated; deacetylation by HDAC4 allows MuRF1 to ubiquitinate MyHC proteins for degradation. This mechanism is consistent with the finding that class IIa HDAC inhibition blocks MuRF1 activity. HDAC4 inhibition increased cellular MyHC content and blocked dexamethasone (DEX)- and denervation-induced protein loss in cells and mice. Moreover, deacetylation of Hsc70 on K128 by HDAC4 enabled Hsc70’s interaction with the glucocorticoid receptor (GR), thereby allowing GR ligand (DEX) binding to promote atrophy. Class IIa HDAC inhibition prevents DEX-induced atrophy. Another study by Renzini *et al.* (26). demonstrated that cytoplasmic HDAC4 plays a protective role in dystrophic muscle by promoting muscle repair, likely by stabilizing Trim72 mRNA. Trim72 deletion in skeletal muscles worsens the pathological features of Duchenne muscular dystrophy (DMD), leading to greater muscle fragility and degeneration over time. The worse phenotype is fully rescued by restoring cytoplasmic levels of HDAC4, both *in vitro* and *in vivo*. These findings suggest that, in the context of muscular dystrophy, enhancing—rather than inhibiting—the cytoplasmic functions of HDAC4 may be beneficial. Therefore, the function of cytoplasmic HDAC4 may depend on the nature of its cytoplasmic substrate. In different muscle atrophy models, cytoplasmic HDAC4 may influence muscle homeostasis by targeting distinct sets of substrates. Nuclear HDAC4 has been reported to induce muscle atrophy in various models of neural injury and disuse, including denervation (28, 29, 30), spinal muscular atrophy (SMA) (31), and immobilization (32, 33) through shared and unique mechanisms. Nuclear accumulation of HDAC4 leads to the upregulation of Myogenin and MyoD expression in SMA (31), denervated (30) and immobilized muscles (32, 33), which are myogenic differentiation factors that activate atrogin-1- and MuRF1-induced protein degradation. In addition to this well-established HDAC4 4/Myogenin positive feedback loop, an additional action mode of HDAC4 is involved in denervation-induced muscle atrophy. Liu *et al.* (29) demonstrated that ALKBH5-mediated m^6^A demethylation stabilizes and increases HDAC4 expression in denervated muscles. HDAC4 interacts with FoxO3 and inhibits its degradation in a deacetylation-dependent manner, ultimately leading to an increase in FoxO3 protein levels and pro-atrophic transcriptional activities. Targeting ALKBH5 reduces HDAC4 levels, which in turn suppresses FoxO3 signaling activation and prevents denervation-induced muscle atrophy. In our ongoing work, we aim to identify the potential substrates of HDAC4 in our muscle atrophy model. Our additional data show that cytoplasmic accumulation of HDAC4 reduces the expression of ER stress markers. Notably, a previous study has reported that cytoplasmic HDAC4 binds to ATF4 and retains it in the cytoplasm of muscle cells, thereby preventing its nuclear translocation and subsequent induction of ER stress and apoptosis (34). These findings suggest that ATF4 is a likely substrate of HDAC4 in our model. This is consistent with our hypothesis that the subcellular localization of HDAC4 substrates underlies the compartment-specific effects of HDAC4 in muscle atrophy. In our study, knockdown of cytoplasmic HDAC4 in Fgf21-deficient mice abolished the protection against denervation-induced muscle atrophy, indicating that cytoplasmic HDAC4 is a key mediator of the beneficial effects associated with Fgf21 deficiency. Given HDAC4’s enzymatic nature, it may interact with cytoplasmic substrates to facilitate NMJ maintenance, which warrants further investigation.

TGFB1 exerts diverse effects *via* TGFB receptors and downstream transcription factors or kinases. Although TGFB1 is known to promote denervation-induced muscle atrophy (35), its interaction with FGF21 has not been studied. Here, we showed that Tgfb1 expression increases specifically in denervated muscle, alongside Fgf21, suggesting that TGFB1 locally induces FGF21 and initiates downstream events. We further demonstrated that TGFB1 activates JNK/c-Jun signaling in mature myotubes to induce FGF21, contributing to NMJ instability and muscle atrophy. Elevated TGFB signaling in ALS models (36), along with FGF21's detrimental effects on NMJs, suggests that the TGFB1/FGF21 axis may underlie neuromuscular degeneration—a hypothesis for future study. As a secreted cytokine, TGFB1 likely originates from intramuscular cells that are activated by denervation. Madaro *et al.* showed that FAPs from denervated—but not injured—muscles promote muscle atrophy (37), and Kajabadi *et al.* reported that cachexia-associated FAPs secrete Activin-A, a TGFB superfamily member, to drive atrophy (38). Based on these findings, we speculated that denervated FAPs might secrete TGFB1 to induce FGF21 and drive muscle atrophy. Supporting this, TGFB1 expression was markedly higher in denervated than in non-denervated FAPs and correlated with their atrophy-inducing ability. Moreover, the FAP marker PDGFRα is upregulated with TGFB1 in ALS muscle, suggesting that FAP-derived TGFB signaling is a promising therapeutic target for muscle atrophy in denervation and ALS contexts.

In summary, we provide the first direct evidence that FGF21 is a critical regulator of neurogenic skeletal muscle atrophy. Through both gain- and loss-of-function approaches, we revealed a novel role for FGF21 in controlling skeletal muscle homeostasis by modulating NMJ innervation. These findings enhance our understanding of muscle homeostasis and highlight the need for cautious evaluation of FGF21 agonists due to potential tissue-specific adverse effects.

## Experimental procedures

### Animals

All mice used in our experiments were housed in pathogen-free conditions under a standard 12:12-h light/dark cycle, with ad libitum access to a standard chow diet (Labo MR Stock, Nosan Corporation Bio-Department) and water. Wilde-type C57BL/6J (WT), mdx (C57BL/10ScSn-Dmdmdx/J) and control mice (C57BL/10ScSn) were purchased from Crea-Japan Inc. Aged (24 months old) and young mice (3 months old) were generously provided by Dr Ishigami (Tokyo Metropolitan Institute for Geriatrics and Gerontology, Japan). Fgf21 global knockout (Fgf21KO) mice were kindly provided by Dr Nobuyuki Itoh (Kyoto University, Japan). The genotypes of the mice were determined as previously described (39).

### Animal models of muscle atrophy

Several muscle atrophy models were employed, including starvation, denervation, muscular dystrophy, and aging. Mice were randomly assigned to experimental groups at the time of surgery/experiment, ensuring that group allocation was unbiased with respect to body weight, age, and other characteristics. In the starvation experiments, animals were deprived of food for 24 or 48 h before being sacrificed. For denervation, one limb of the mouse was shaved and sterilized, the sciatic nerve was exposed, and a 5 to 10 mm segment was excised. The incision was then sutured using an 8-0 sterile silk suture. The contralateral limb, which was subjected to the same operation without sciatic nerve transection, served as the sham group. Different denervation strategies were selected based on the experimental objectives. For general biological experiments (*e.g.*, gene expression, protein expression, and histological analysis) and *in vivo* HDAC4 knockdown studies, unilateral sham surgery and contralateral denervation were performed on the same mice. For grip strength tests and evaluation of Fgf21 expression across different tissues, both limbs of a mouse were either sham-operated or denervated separately. In experiments involving *in vivo* FGF21 overexpression or ELISA assays of plasma, one limb of a mouse was either sham-operated or denervated, respectively. Mice were sacrificed at 3-, 7-, 14-, or 31-days post-surgery. The muscular dystrophy and aging models directly utilized mdx mice and aged mice, respectively. Muscles collected at the specified times were either prepared for histological analysis or frozen in liquid nitrogen and stored at −80 °C for future use. Although analyses were not performed under blinded conditions due to practical constraints (*e.g.*, single-operator procedures), objective criteria and automated image quantification were applied to minimize bias.

### AAV constructs, production, and purification

The AAV2/8-TBG-GFP expression plasmid, p5E18-VD2/8 helper plasmid, and pXX6-80 capsid plasmid were generously provided by Dr Itoh (40). AAV serotype 8 is known for its tropism for skeletal muscle cells (41). To drive *in vivo* muscle-specific gene expression, the 1350 base pair region of the pBS MCK promoter (Addgene, #12528), which has been shown to efficiently and specifically drive gene expression in skeletal muscle (42), was amplified and used to replace the TBG promoter of AAV2/8-TBG-GFP using PacI/EcoRI restriction enzyme sites, resulting in the AAV2/8-MCK-GFP plasmid. To achieve *in vivo* gene knockdown, the GFP-U6 expression cassettes were excised from the AAV2/1-GFP-U6 plasmid, a gift from Dr Eguchi (43), and used to replace the TBG promoter and GFP of AAV2/8-TBG-GFP using PacI/HindIII restriction enzyme sites, to generate the AAV2/8-GFP-U6 plasmid. To overexpress FGF21, the coding sequence for mouse Fgf21 was inserted to replace the Gfp sequence in AAV2/8-MCK-GFP, with AAV2/8-MCK-GFP serving as a control virus. To reduce HDAC4 expression, two shRNA sequences targeting mouse Hdac4 (44, 45) or a non-targeting control sequence (46) were inserted into the AAV2/8-GFP-U6 plasmid (resulting in AAV2/8-GFP-shHDAC4 and AAV2/8-GFP-shCtrl, respectively). These constructs were placed under the control of the U6 promoter, with GFP serving as a reporter to monitor shRNA delivery efficiency. AAV vectors used for overexpression or knockdown experiments were packaged by triple transfection of expression plasmids, helper plasmids, and capsid plasmids into HEK293T cells, followed by purification *via* CsCl density gradient ultracentrifugation (47). Viral titers were determined using TaqMan real-time quantitative PCR. The shRNA sequences are shown in Table S1.

### AAV injections in skeletal muscles

For overexpression experiments, 5-week-old male C57BL/6J mice were intramuscularly injected with 2.5 × 10^10^ viral genomes (vg) of either AAV2/8-MCK-GFP or AAV2/8-MCK-mFGF21 vectors into the tibialis anterior (TA) muscles. For knockdown experiments, 5-week-old male C57BL/6J mice were intramuscularly injected with 1 × 10^11^ vg of either AAV2/8-GFP-shCtrl or AAV2/8-GFP-shHDAC4 vectors into TA muscles. Four weeks post-injection, denervation surgery was performed as previously described.

### Histological analysis

TA muscle samples were snap-frozen in liquid nitrogen-precooled isopentane, embedded in Tissue-Tek O.C.T. compound (Sakura Finetek), and sectioned into 10 μm cryosections. For Hematoxylin and Eosin (H&E) staining, cryosections were air-dried, fixed in 4% paraformaldehyde, washed in PBS, and stained with hematoxylin and eosin following standard protocols. The cross-sectional area was measured using Fiji software, as described in a published protocol (48).

### Grip strength

A grip strength meter (Muromachi, MK-380V) was used to measure grip strength. The mice were allowed to grasp the metal grid of the device with all four paws and were gently pulled backward by their tails in a straight line until the grip was broken and the peak force measurement was recorded. Each mouse was tested five times with 30-s intervals between tests. The highest and lowest values were excluded, and the remaining three force measurements were averaged and normalized to body weight.

### Subcellular fractionation

Nuclear protein extraction from tissues was performed according to a previously published protocol (49). Briefly, 50 mg of TA muscle was homogenized in STM buffer (250 mM sucrose, 50 mM Tris–HCl pH 7.4, 5 mM MgCl2), and the supernatant was precipitated using four times the volume of pre-chilled acetone. After acetone was completely removed, the resulting pellet was resuspended and used as the cytosolic fraction. For the nuclear fraction, the pellet obtained in the first step was washed three times in STM buffer and then resuspended in NET buffer (20 mM HEPES pH 7.9, 1.5 mM MgCl2, 0.5 M NaCl, 0.2 mM EDTA, 20% glycerol, 1% Triton X-100). The suspension was then passed through an 18-gauge needle, and the supernatant was collected as the nuclear fraction. All procedures were performed on ice, and protease and phosphatase inhibitor cocktails were added to each buffer immediately before use.

### Whole-mount neuromuscular junction (NMJ) staining

The protocol for NMJ staining of extensor digitorum longus (EDL) muscles was adapted from a published method (50). EDL muscles were dissected and fixed in 4% paraformaldehyde (PFA) in PBS, then dehydrated in 30% sucrose. After rinsing with PBS, muscles were incubated in 0.1 M glycine to quench formaldehyde-induced autofluorescence. The muscles were then separated into four pieces and permeabilized in a solution containing 0.5% Triton X-100 and 4% BSA, followed by incubation with primary antibodies: mouse anti-NF-M (1:200, 2H3, DSHB) and mouse anti-SV2 (1:200, 2H3, DSHB) in a blocking solution containing 2% Triton X-100 and 4% BSA. After washing with 2% Triton X-100 in PBS, the muscles were incubated with secondary antibody Alexa Fluor 488 (1:500, A11029, Invitrogen) and α-BTX (1:500, Invitrogen) for 2 h. The muscles were then washed again with 2% Triton X-100 in PBS, mounted with ProLong Glass Antifade with NucBlue (Thermo Fisher Scientific), flattened with magnets, and sealed with nail polish. Samples were stored in the dark until imaging with a ZEISS LSM 800 confocal laser scanning microscope (Carl Zeiss).

### Cell isolation

Murine primary cells from denervated mice were isolated using magnetic-activated cell sorting (MACS, Miltenyi). Briefly, all hindlimb skeletal muscles were dissected, mechanically dissociated, and digested in an enzymatic mix containing 2 mg/ml collagenase D, 2.4 U/ml dispase II, and 10 μg/ml DNase I, supplemented with 1 mM CaCl2 and 5 mM MgCl2. The digested muscles were then passed through an 18-gauge needle several times. The resulting mixture was sequentially filtered through 100, 70, and 40 μm cell strainers and incubated with RBC lysis buffer to remove erythrocytes. Different cell populations were then isolated through consecutive incubation with microbead-conjugated antibodies in the cell suspension. Lin+/CD31+ cells were collected as lineage cells and endothelial cells. Fibro/adipogenic progenitors (FAPs) were defined as Lin-/CD31-/α7-integrin-, PDGFRα+ cells.

### Fibro/adipogenic progenitors (FAPs) culture and conditioned medium (CM) collection

Freshly isolated FAPs were cultured in Dulbecco’s modified Eagle’s medium (DMEM) containing 20% FBS until they reached confluence. The cells were then washed with PBS and cultured in serum-free DMEM for 24 h. The conditioned medium (CM) was collected and centrifuged at 3000 rpm for 10 min at 4 °C, and the supernatant was stored at −80 °C until use. CM treatments were applied to 3-days differentiated C2C12 cells for the specified periods.

### RNA sequencing (RNA-seq) and public datasets analysis

Two weeks post-denervation, TA muscles from sham-operated and denervated mice (n = 4 per group) were collected. Total RNA was extracted using the RNeasy kit (QIAGEN), and the RNA from each group was pooled and submitted to Macrogen Co., Ltd for analysis. Differentially expressed genes (DEGs) were analyzed using the edgeR software package, with DEGs identified based on an adjusted *p*-value < 0.05 and |logFC| ≥ 1. Data analysis and visualization were performed in R (v 4.3.1). Upregulated genes used to generate a Venn diagram were obtained from the NCBI Gene Expression Omnibus (GEO) datasets: GSE18119 (mouse quadriceps muscle, mitochondrial myopathy) (unpublished), GSE48574 (human skeletal muscle biopsies, iron-sulfur [Fe-S] cluster-deficient myopathy) (51), GSE52766 (mouse gastrocnemius muscle, mdx) (52), GSE49826 (mouse TA muscle, 2-weeks denervation) (53), and GSE87108 (mouse gastrocnemius muscle, 28-month-old) (54). The screening threshold was set at *p* < 0.05. Additionally, the expression of Fgf21 in denervated TA muscles of rats was analyzed using GSE201025 (rat TA muscle, 2-weeks denervation) (55).

### Plasmid constructs

The coding sequence of mouse Hdac4 was cloned into a p3XFLAG-CMV-7.1 vector (Sigma-Aldrich) using HindIII and EcoRI restriction enzyme sites to construct the FLAG-Hdac4 expression plasmid.

### Cell culture

Murine myoblasts C2C12 (obtained from ATCC) were maintained at 37 °C and 5% CO2 in high-glucose DMEM supplemented with 10% FBS. Cells were sub-cultured when they reached 50 to 60% confluence. Once confluent, the culture medium was replaced with a differentiation medium, consisting of high-glucose DMEM supplemented with 2% horse serum, to induce myotube formation. Fresh differentiation medium was replenished every 2 days, and cells were allowed to differentiate for 3 to 4 days before experiments. The passage number of C2C12 cells used in this research did not exceed 20.

### RNA interference

siRNAs targeting Fgf21, Smad3, and control siRNA were purchased from Dharmacon (ON-TARGETplus Mouse Fgf21 [56636] siRNA–SMARTpool, ON-TARGETplus Mouse Smad3 [17127] siRNA–SMARTpool, and ON-TARGETplus Non-targeting pool). C2C12 cells that had been differentiated for 2 days were transfected with control siRNA (10 or 30 nM), or siRNA targeting mouse Fgf21 (10 nM) or Smad3 (30 nM) using Lipofectamine RNAiMAX Transfection Reagent (Thermo Fisher) according to the manufacturer’s instructions. 24 or 48 h after transfection, cells were used for the indicated experiments as detailed in the Results section. The siRNA sequences are shown in Table S2.

### Immunofluorescence (IF) staining

Myotube diameter and the localization of exogenous or endogenous HDAC4 were determined by confocal microscopy following a standard immunofluorescence (IF) staining protocol. C2C12 cells were washed, fixed with 4% paraformaldehyde (PFA), permeabilized with 0.5% Triton X-100, and blocked with 3% BSA. The specimens were then incubated with anti-MyHC (MF20; R&D Systems), anti-HDAC4 (CST), or anti-FLAG (Sigma) antibodies for 2 h, followed by incubation with Alexa Fluor 488–conjugated goat anti-mouse or anti-rabbit IgG antibodies (1:500) for 1 h. The specimens were mounted with ProLong Glass Antifade with NucBlue (Thermo Fisher Scientific) and observed using a ZEISS LSM800 confocal laser scanning microscope (Carl Zeiss).

### Acetylcholine receptor (AChR) clustering assay

The stability of AChR clusters was assessed as previously described (56). Briefly, after 4 days of differentiation to induce AChR clusters, C2C12 myotubes were treated with agrin alone or in combination with other compounds for 16 to 18 h and then maintained in a medium with or without additional compounds. Cells were fixed in 2% PFA, and AChRs were stained with α-bungarotoxin 594 (BTX; 1 mg/ml, Invitrogen) diluted 1:2500. After washing, cells were mounted with ProLong Glass Antifade with NucBlue (Thermo Fisher Scientific). Samples were stored and protected from light until imaging with a ZEISS LSM 800 confocal laser scanning microscope (Carl Zeiss).

### Immunoblotting

C2C12 cells and mouse tissues were lysed in RIPA buffer containing PMSF (Sigma-Aldrich, MO), a protease inhibitor cocktail (Nacalai Tesque), and a phosphatase inhibitor cocktail (Sigma-Aldrich). The protein lysates were then boiled and subjected to SDS-PAGE, followed by incubation with primary and secondary antibodies according to standard procedures. The antibodies used are shown in Table S3.

### Quantitative real-time PCR (RT-qPCR)

Total RNA from cells or mouse tissues was extracted using ISOGEN (NIPPON GENE) according to the manufacturer’s instructions and reverse transcribed into cDNA using the High-Capacity cDNA Reverse Transcription Kit (Applied Biosystems). Quantitative real-time PCR was performed on an Applied Biosystems StepOnePlus instrument using SYBR Green Master Mix (Thermo Fisher Scientific). The qPCR primers used are shown in Table S4.

### Enzyme-linked immunosorbent assay (ELISA)

The protein levels of FGF21 and TGFB1 in the plasma, muscle tissue, and culture medium were measured using a Mouse/Rat FGF-21 ELISA kit (R&D Systems) or a Human/Mouse/Rat/Porcine/Canine TGF-beta 1 ELISA kit (R&D Systems), according to the manufacturer’s instructions.

### Statistical analysis

Data are presented as mean ± SD from at least three independent biological replicates. All experiments were repeated on at least two occasions with similar results. A two-tailed Student’s *t* test was used for comparisons between the two groups. For comparisons involving more than two groups, one-way analysis of variance (ANOVA) followed by Tukey’s multiple comparison tests or two-way ANOVA followed by Sidak’s multiple comparison test was applied. Post hoc power analyses were conducted using G∗Power 3.1 for the relevant two-way ANOVA or *t* test. The effect sizes (Cohen’s f or d) and achieved power (1–β) are reported in Table S3. Statistical significance is denoted in the figures as ∗(*p* < 0.05), ∗∗(*p* < 0.01), and ∗∗∗(*p* < 0.001). Statistical analysis was performed using GraphPad Prism 9.0 software.

## Ethics statement

All animal studies were conducted in accordance with the guidelines approved by the Animal Usage Committee of the University of Tokyo (Law No. 105, 1 October 1973, as amended on 1 June 2020).

## Data availability

All data are contained within the manuscript. The dataset generated for the RNA-seq is available through the Gene Expression Omnibus under the accession code: GSE303861.

## Supporting information

This article contains supporting information.

## Conflict of interest

The authors declare that they have no conflicts of interest with the contents of this article.
