# Supplementary material for: The growth factor FGF21 maintains neuromuscular junction through histone deacetylase HDAC4 in denervation-induced skeletal muscle atrophy
Source: J Biol Chem. 2025 Sep 23;301(11):110756. doi: 10.1016/j.jbc.2025.110756 (PMC12554194; doi:10.1016/j.jbc.2025.110756)
Supplement: Supporting Figures and Tables [file mmc1.docx]

**A growth factor regulates neuromuscular junction innervation through histone deacetylase in denervation-induced skeletal muscle atrophy**

Lirong Zheng, Takashi Sasaki, Liyang Ni, Tsutomu Hashidume, Mitsuki Kawabe, Yu Takahashi, Yoshio Yamauchi, Makoto Shimizu, Ryuichiro Sato

Contents in order listed

Supplementary figure 1

Supplementary figure 2

Supplementary figure 3

Supplementary figure 4

Supplementary figure 5

Supplementary figure 6

Supplementary figure 7

Supplementary figure 8

Supplemental table 1

Supplemental table 2

Supplemental table 3

***Supplemental figures***

**
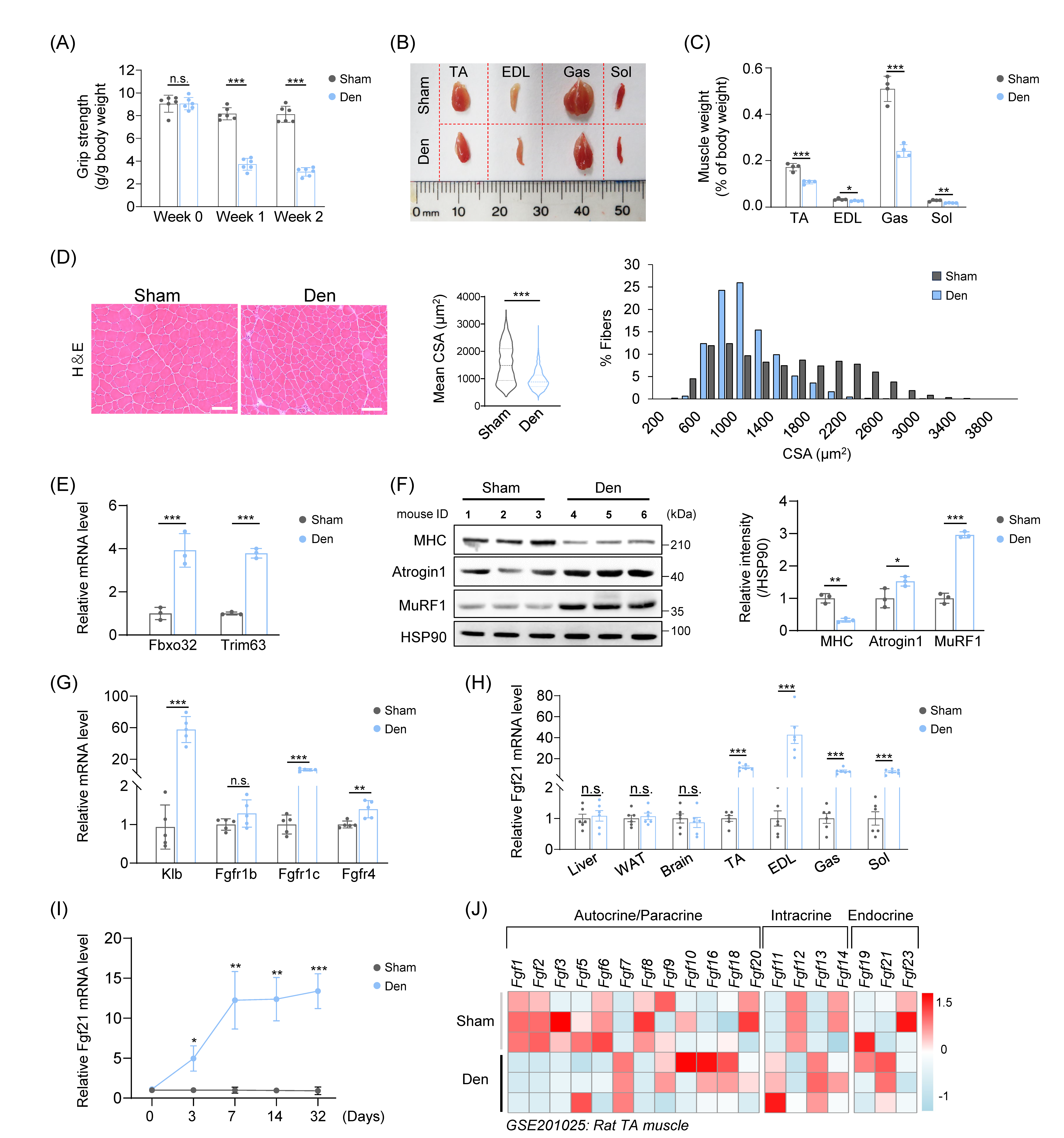
**

**Figure S1. Fgf21 expression pattern in the denervation model**

Mice were subjected to denervation for 2 weeks (except in G, where 3-day denervation was performed).

(A) Four-limb grip strength (g/g body weight) of mice (n = 6).

(B) Representative image of the hindlimb skeletal muscles.

(C) The ratio of limb muscle weight to body weight (n = 4).

(D) Representative cross-sectional images of TA muscle (n = 4). Scale bar, 50 μm.

(E) mRNA levels of Fbxo32 and Trim63 in TA muscle (n = 3).

(F) Protein levels of MHC, Atrogin1, and MuRF1 in TA muscle (n = 3).

(G) mRNA levels of Klb and FGF receptors in TA muscle (n = 5).

(H) mRNA level of Fgf21 in different tissues (n = 6).

(I) mRNA level of Fgf21 at indicated times post-denervation (n = 3-5).

(J) Heatmap showing Fgf21 expression in TA muscle of rats after 2-week denervation (GSE201025, n = 3).

Data were analyzed by two-tailed Student’s t-test. ∗p < 0.05; ∗∗p < 0.01; ∗∗∗p < 0.001.

**
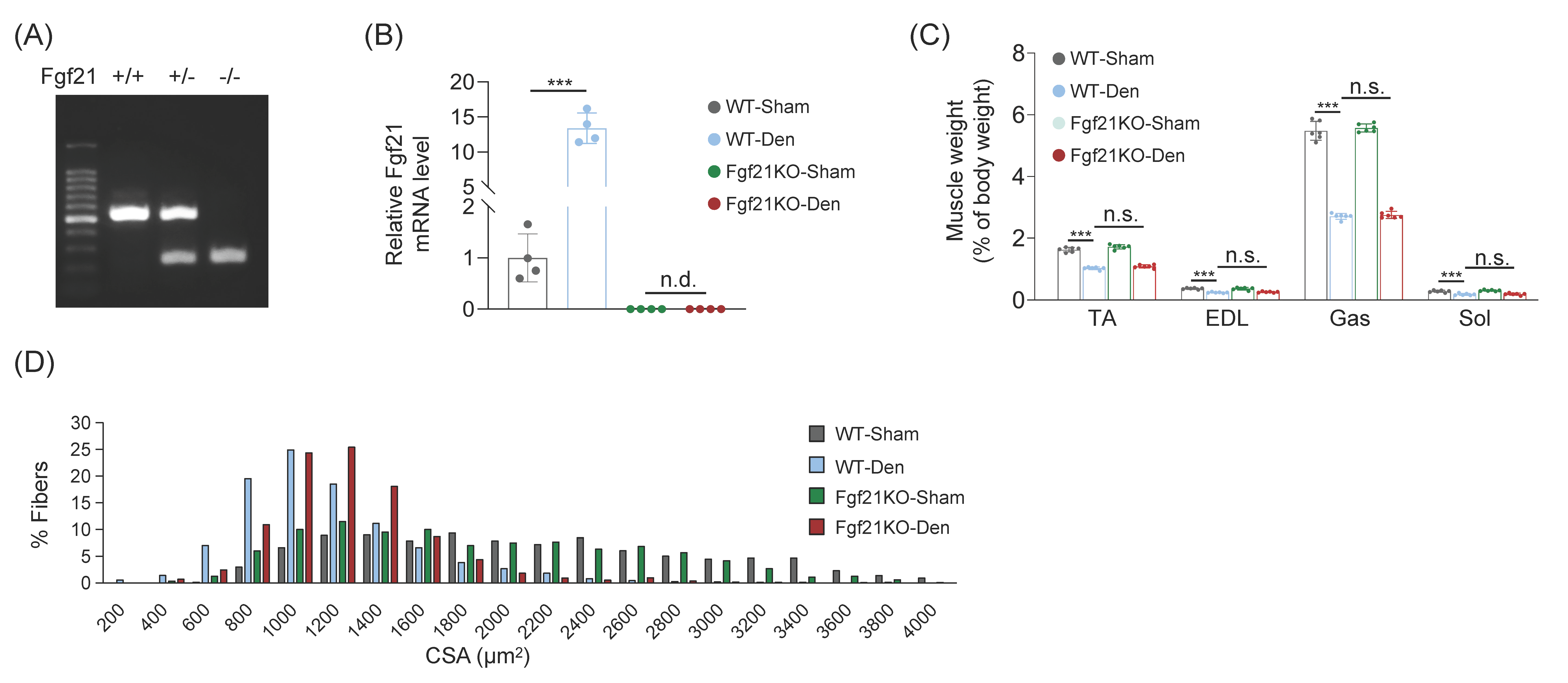
**

**Figure S2. Fgf21 deficiency protects skeletal muscle from denervation-induced atrophy**

(A) Genotyping of Fgf21KO mice.

(B) mRNA levels of Fgf21 in TA muscle from WT and Fgf21KO mice (n = 4).

(C) The ratio of limb muscle weight to body weight of WT and Fgf21KO mice (n = 6).

(D) Fiber size distribution in TA muscle from WT and Fgf21KO mice (n = 4).

Data were analyzed by two-way ANOVA followed by Sidak’s multiple comparisons test. In (B,C), different interactions between genotype and treatment were detected (in (B), F(1,12) = 123.631, p < 0.001; in (C), TA: F(1,20) = 0.484, p = 0.494; EDL: F(1,20) = 0.635, p = 0.435; Gas: F(1,20) = 0.087, p = 0.770; Sol: F(1,20) = 0.566, p = 0.461). ∗p < 0.05; ∗∗p < 0.01; ∗∗∗p < 0.001.



**Figure S3. S3 FGF21 promotes denervation-induced skeletal muscle atrophy.**

(A) mRNA levels of Fgf21 in TA muscle injected with AAV-GFP or AAV-FGF21 (n = 4).

(B) Protein levels of FGF21 and GFP in TA muscle injected with AAV-GFP or AAV-FGF21 (n = 4). The loading control used here is the same as that shown in Figure 5H.

(C) Protein levels of FGF21 in plasma of mice injected with AAV-GFP or AAV-FGF21 (n = 4).

(D) Four-limb grip strength (g/g body weight) of mice injected with AAV-GFP or AAV-FGF21 (n = 7).

(E) The ratio of limb muscle weight to body weight of mice injected with AAV-GFP or AAV-FGF21 (n = 4).

(F) Representative cross-sectional images of TA muscle injected with AAV-GFP or AAV-FGF21 (n = 4). Scale bar, 50 μm.

(G) Protein levels of rescued FGF21 in plasma (n = 5).

(H) The ratio of limb muscle weight to body weight of mice after rescued FGF21 expression (n = 5-6).

(I) Fiber size distribution in TA muscle after rescued FGF21 expression (n = 4).

In (A-F), data were analyzed by two-way ANOVA followed by Sidak’s multiple comparisons test. Different interactions between genotype and virus treatment were detected (in (A), F(1,12) = 14.949, p = 0.002; in (B), FGF21: F(1,12) = 11.366, p = 0.006; GFP: F(1,12) = 0.725, p = 0.411; in (C), F(1,12) = 0.581, p = 0.461; in (D), week 0: F(1,24) = 6.865, p = 0.015; week 1: F(1,24) = 1.170, p = 0.290; week 2: F(1,24) = 0.307, p = 0.585; in (E), TA: F(1,12) = 0.072, p = 0.792; EDL: F(1,12) = 0.295, p = 0.597; Gas: F(1,12) = 0.362, p = 0.559; Sol: F(1,12) = 0.958, p = 0.347; in (F), F(1,5266) = 2.308, p = 0.129). In (G-I), data were analyzed by one-way ANOVA followed by Tukey’s multiple comparisons test. ∗p < 0.05; ∗∗p < 0.01; ∗∗∗p < 0.001.



**Figure S4. Analysis of Fgf21KO skeletal muscle by RNA sequencing**

(A) Heatmap showing gene expression changes in TA muscle from WT and Fgf21KO mice after 2-week denervation (4 samples were pooled for each group).

(B) Venn diagram showing the overlap of differentially expressed genes (DEGs) across four comparison groups.

(C) Volcano plot of DEGs in each comparison group.

(D) Gene Ontology (GO) enrichment analysis of DEGs.

WS, WT-Sham; WD, WT-Denervation; KS, Fgf21KO-Sham; KD, Fgf21KO-Denervation.





**Figure S5. Fgf21 deficiency resists muscle atrophy via HDAC4**

(A) Protein levels of HDAC5 and HDAC7 in TA muscle from WT and Fgf21KO mice (n = 3).

(B) Protein levels of HDAC4 in sham-operated TA muscle injected with shRNA against Hdac4 or scramble control (n = 3-4).

(C) Representative cross-sectional images of sham-operated TA muscle following HDAC4 knockdown (n = 5). Scale bar, 50 μm.

(D) Fiber size distribution of denervated TA muscle following HDAC4 knockdown (n = 5).

(E) Representative images of NMJ innervation in sham-operated EDL muscle following HDAC4 knockdown (n = 4).

Data were analyzed by two-way ANOVA followed by Sidak’s multiple comparisons test. Different interactions between genotype and treatment were detected (in (A), HDAC5: F(1,8) = 0.382, p = 0.554; HDAC7: F(1,8) = 0.159, p = 0.701; in (B), #1: F(1,9) = 0.391, p = 0.547; #2: F(1,9) = 7.261, p = 0.025; in (C), #1: F(1,3359) = 20.211, p < 0.001; #2: F(1,3141) = 9.589, p = 0.002; in (E), #1: F(1,12) = 0.560, p = 0.469; #2: F(1,12) = 1.579, p = 0.233). ∗p < 0.05; ∗∗p < 0.01; ∗∗∗p < 0.001.


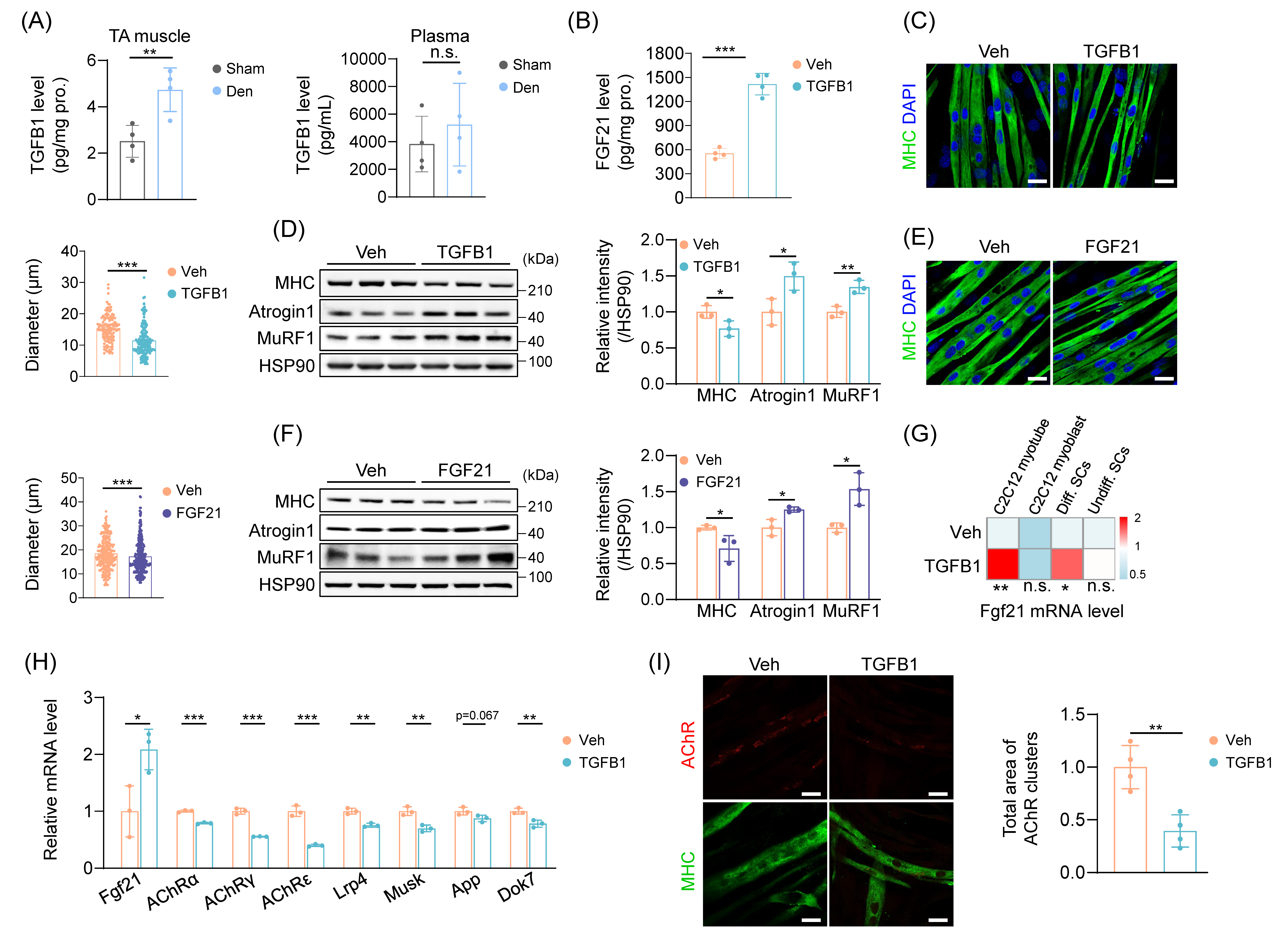


**Figure S6. TGFB1 promotes myotube atrophy via FGF21-mediated NMJ damage**

(A) Protein levels of TGFB1 in TA muscle and plasma (n=4).

(B) Protein levels of FGF21 in C2C12 culture medium treated with TGFB1 (n = 4).

(C) Representative images of C2C12 myotube diameter treated with TGFB1 (n = 3). Scale bar, 20 μm.

(D) Protein levels of MHC, Atrogin1, and MuRF1 in C2C12 myotubes treated with TGFB1 (n = 3).

(E) Representative images of C2C12 myotube diameter treated with FGF21 (n = 3). Scale bar, 20 μm.

(F) Protein levels of MHC, Atrogin1, and MuRF1 in C2C12 myotubes treated with FGF21 (n = 3).

(G) Heatmap showing Fgf21 mRNA levels in different cell types treated with TGFB1 (n = 3).

(H,I) mRNA levels of NMJ-related genes (n = 3) and representative images of AChR intensity in C2C12 myotubes treated with TGFB1 (n = 4). Scale bar, 20 μm.

Diff. SCs: differentiated satellite cells; Undiff. SCs: Undifferentiated satellite cells.

Data were analyzed by two-tailed Student’s t-test. ∗p < 0.05; ∗∗p < 0.01; ∗∗∗p < 0.001.


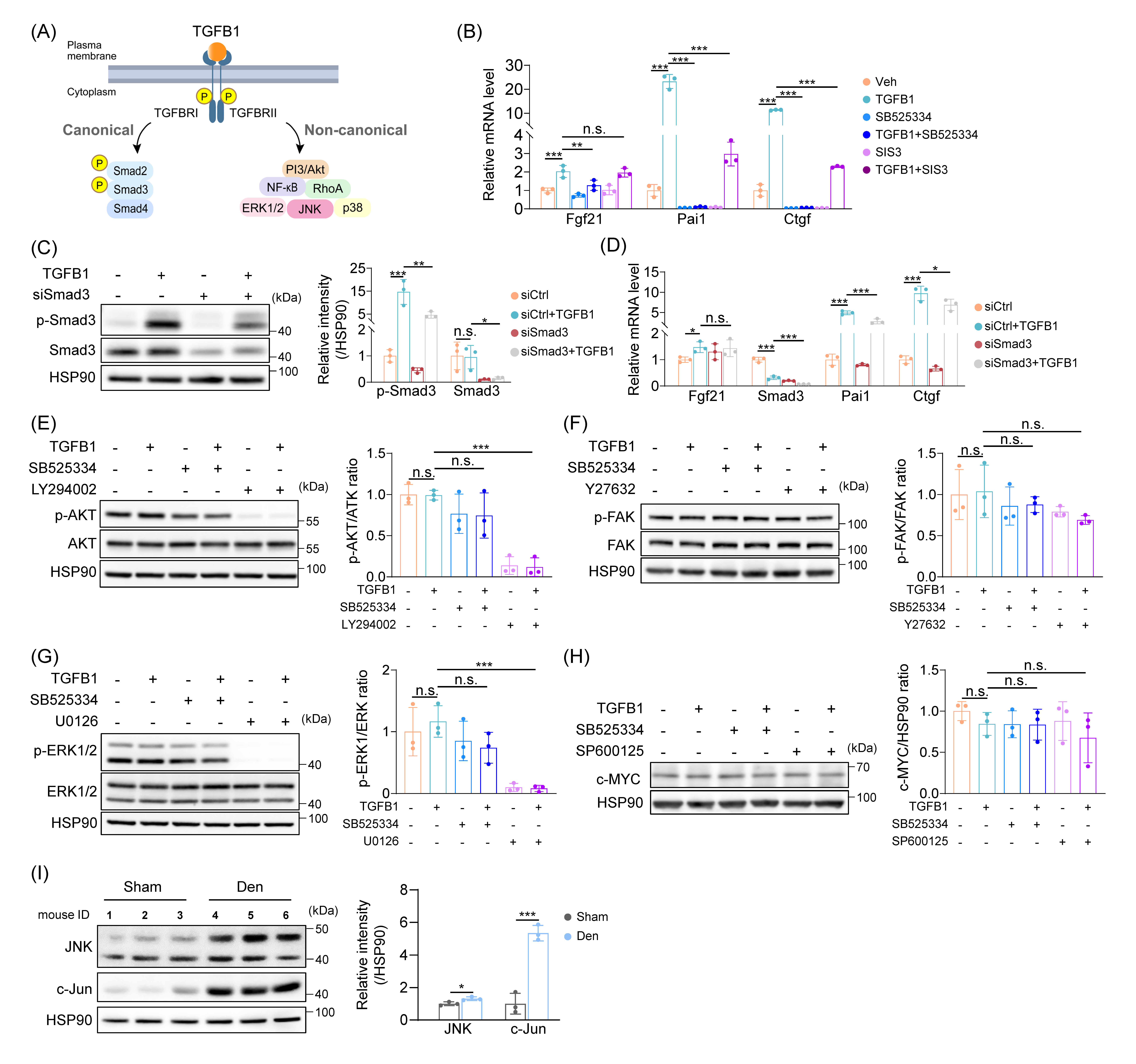


**Figure S7. Fgf21 is not regulated by canonical TGFB signaling**

(A) Schematic of TGFB signaling.

(B) mRNA levels of Fgf21, Pai1, and Ctgf in C2C12 myotubes treated with TGFB1, SB525334 and SIS3 (n = 3).

(C) Protein levels of Smad3 in in C2C12 myotubes treated with TGFB1 and siSmad3 (n=3).

(D) mRNA levels of Fgf21, Smad3, Pai1, and Ctgf in C2C12 myotubes treated with TGFB1, SB525334 and respect (n = 3).

(E-H) Protein levels of AKT, FAK, ERK and c-MYC in C2C12 myotubes treated with TGFB1, SB525334 and corresponding inhibitors (n = 3).

(I) Protein levels of JNK and c-Jun protein in TA muscle (n = 3).

In (B-H), data were analyzed by two-way ANOVA followed by Sidak’s multiple comparisons test. Different interactions between genotype and treatment were detected (in (B), Fgf21: F(1,12) = 1.796, p = 0.208; Pai1: F(1,12) = 144.060, p < 0.001; Ctgf: F(1,12) = 1828.294, p < 0.001; in (C), p-Smad3: F(1,8) = 8.478, p = 0.020; Smad3: F(1,8) = 0.058, p = 0.816; in (D), Fgf21: F(1,8) = 1.413, p = 0.269; Smad3: F(1,8) = 72.510, p < 0.001; Pai1: F(1,8) = 16.945, p = 0.003; Ctgf: F(1,8) = 3.568, p = 0.096; in (E), F(1,12) = 0.003, p = 0.997; in (F), F(1,12) = 0.189, p = 0.830; in (G), F(1,12) = 0.459, p = 0.643; in (H), F(1,12) = 0.401, p = 0.678). In (I), data were analyzed by two-tailed Student’s t-test. ∗p < 0.05; ∗∗p < 0.01; ∗∗∗p < 0.001.


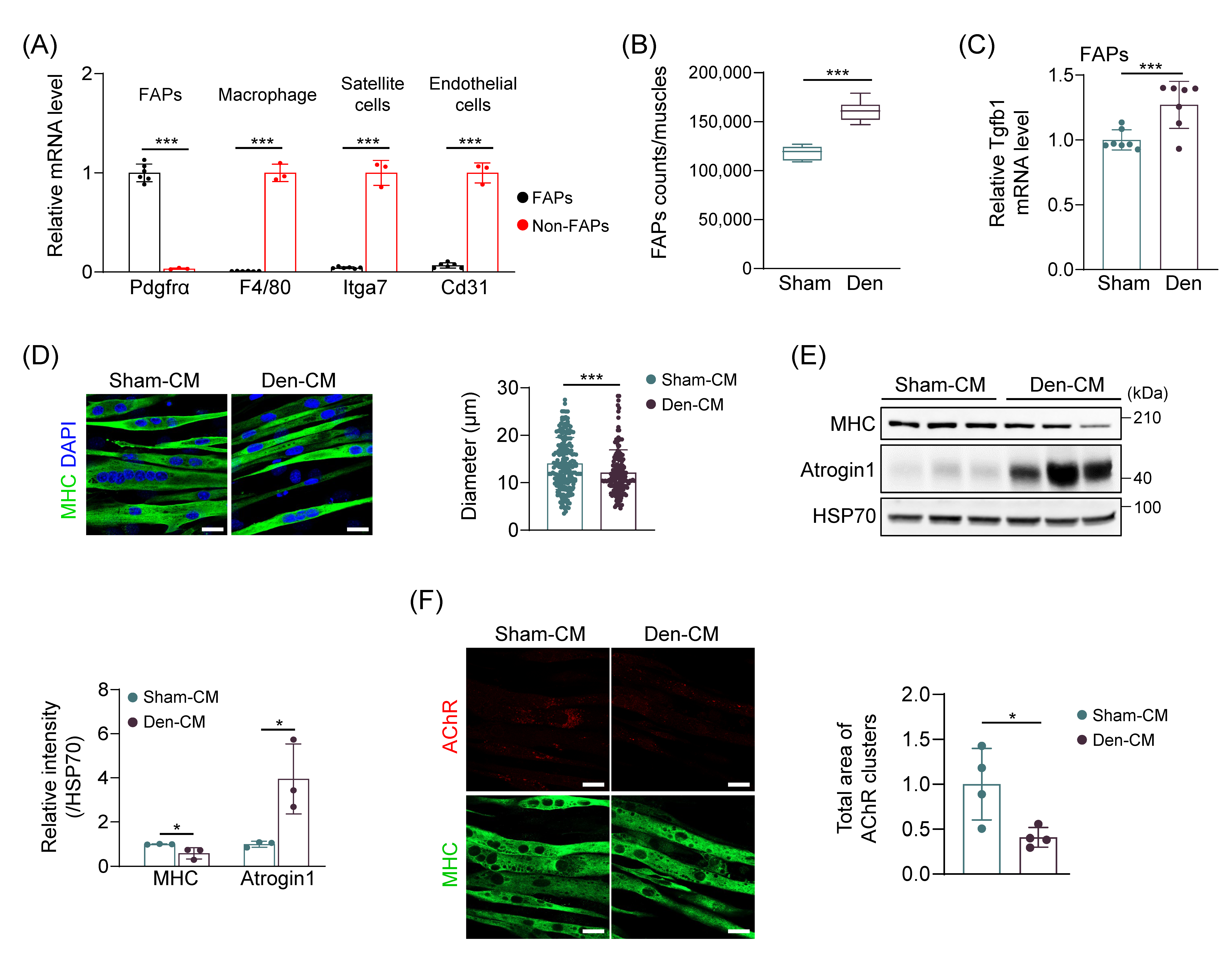


**Figure S8. FAPs-derived conditioned medium (CM) promotes muscle atrophy**

(A) mRNA levels of Pdfgrα, F4/80, Itga7, and Cd31 in MACS-isolated FAPs and non-FAPs fractions (n = 3-6).

(B) Numbers of FAPs extracted from limb skeletal muscles (n = 7).

(C) mRNA levels of Tgfb1 in FAPs (n = 7).

(D) Representative images of C2C12 myotube diameter treated with conditioned medium (CM) (n = 3). Scale bar, 20 μm.

(E,F) Protein levels of MHC and Atrogin1 (n=3) and representative images of AChR intensity in C2C12 myotubes treated CM (n=4). Scale bar, 20 μm.

Data were analyzed by two-tailed Student’s t-test. ∗p < 0.05; ∗∗∗p < 0.001.

**Table S1. List of antibodies used for Western blot and Immunofluorescence.**

| **Antibodies** | **Source** | **Identifier** |
| --- | --- | --- |
| Anti-MHC (MF20) | R&D Systems | MAB4470 |
| Anti-Fbx32 (Atrogin1) | Abcam | ab168372 |
| Anti-MuRF1 | Santa Cruz Biotechnology | sc-398608 |
| Anti-HSP90 | Santa Cruz Biotechnology | sc-13119 |
| Anti-FGF21 | Abcam | ab171941 |
| Anti-FGF21 | R&D Systems | AF3057 |
| Anti-GFP | Abcam | ab6566 |
| Anti-β-actin | Sigma-Aldrich | A5441 |
| Anti-α-tubulin | Cell Signaling Technology | #2144 |
| Anti-TGFB1 | Abcam | ab64715 |
| Anti-phospho-Smad3 | Abcam | ab52903 |
| Anti-Smad3 | Cell Signaling Technology | #9523 |
| Anti-phospho-c-Jun | Cell Signaling Technology | #9261 |
| Anti-c-Jun | Cell Signaling Technology | #9165 |
| Anti-phospho-SAPK/JNK | Cell Signaling Technology | #9251 |
| Anti-SAPK/JNK | Cell Signaling Technology | #9252 |
| Anti-phospho-Akt | Cell Signaling Technology | #9271 |
| Anti-Akt | Cell Signaling Technology | #9273 |
| Anti-phospho-FAK | Cell Signaling Technology | #3283 |
| Anti-FAK | Cell Signaling Technology | #3285 |
| Anti-phospho-ERK1/2 | Cell Signaling Technology | #9101 |
| Anti-ERK1/2 | Cell Signaling Technology | #9102 |
| Anti-c-Myc | Cell Signaling Technology | #2278 |
| Anti-HSP70 | Santa Cruz | sc-32239 |
| Anti-HSP90 | Santa Cruz | sc-13119 |
| Anti-LC3 | Cell Signaling Technology | #12741 |
| Anti-NF-M | Developmental Studies Hybridoma Bank | 2H3 |
| Anti-SV2 | Developmental Studies Hybridoma Bank | SV2 |
| Anti-HDAC4 | Cell Signaling Technology | #15164 |
| Anti-HDAC5 | Cell Signaling Technology | #20458 |
| Anti-HDAC7 | Cell Signaling Technology | #33418 |
| Anti-GAPDH | Cell Signaling Technology | #2118 |
| Anti-phospho-HDAC4/5/7(S246/S259/S155) | Cell Signaling Technology | #3443 |
| Anti-phospho-HDAC4/5/7(S632/S661/S486) | Cell Signaling Technology | #3424 |
| Anti-phospho-AMPK | Cell Signaling Technology | #2535 |
| Anti-AMPK | Cell Signaling Technology | #2532 |
| Anti-FLAG-M2 | Sigma-Aldrich | F3165 |
| Anti-Lamin A/C | Cell Signaling Technology | #2032 |
| Anti-Histone H3 | Cell Signaling Technology | #4499 |

**Table S2. List of primers used for qRT-PCR analysis.**

| **Gene** | **Forward (5' -> 3')** | **Reverse (5' -> 3')** |
| --- | --- | --- |
| 18S | ACCGCAGCTAGGAATAATGGA | GCCTCAGTTCCGAAAACCA |
| Fgf1 | ACACCGAAGGGCTTTTATACG | GTGTAAGTGTTATAATGGTTTTCTTCCA |
| Fgf2 | CAACCGGTACCTTGCTATGA | TCCGTGACCGGTAAGTATTG |
| Fgf3 | ACGGCAGCCTTGAGAACA | CCACTTCCACCGCAGTAATC |
| Fgf4 | CGGCTCTACTGCAACGTG | CGGAGAGAGCTCCAGAAGAC |
| Fgf5 | CTGCAGATCTACCCGGATG | TCCTCGTATTCCTACAATCCC |
| Fgf6 | CTGTACACAACGCCCAGCTT | TTGTTTGGAAGGAGGGTTTCTC |
| Fgf7 | AAGGGACCCAGGAGATGAAG | ACTGCCACGGTCCTGATTT |
| Fgf8 | CATGGCAGAAGACGGAGAC | ACTCGGACTCTGCTTCCAAA |
| Fgf9 | CTATCCAGGGAACCAGGAAAGA | CAGGCCCACTGCTATACTGATAAA |
| Fgf10 | GCGGGACCAAGAATGAAGA | AGTTGCTGTTGATGGCTTTGA |
| Fgf11 | TTGTACAGCTCGCCACATTTC | GTAATTCTCAAAGACGCACTCCTT |
| Fgf12 | CATTTTGTACCAAAACCTATTGAAGTG | TCCTTGAGCGTCCTTGCTT |
| Fgf13 | AATGAACAGCGAGGGATACTTG | ACTGATTCTTTGAATTTGCACTCA |
| Fgf14 | CCCGATGGAGCTCTCGAT | GGTTGAACAGTGTGGAATTGGT |
| Fgf15 | ACGGGCTGATTCGCTACTC | TGTAGCCTAAACAGTCCATTTCCT |
| Fgf16 | GGCCTGTACCTAGGAATGAATGA | TTCCCGGAAAACACATTCAC |
| Fgf17 | GGCAAATCCGTGAATACCA | CTGCTGCCGAATGTATCTGT |
| Fgf18 | TGCTGTGCTTCCAGGTTCA | GGATGCGGAAGTCCACATT |
| Fgf20 | CGGCAGGATCACAGTCTCTT | CCAGTCCCACTGCCACACT |
| Fgf21 | CCTCTAGGTTTCTTTGCCAACAG | AAGCTGCAGGCCTCAGGAT |
| Fgf22 | GTGGGCACTGTGGTGATCA | GCGATTCATGGCCACATAGA |
| Fgf23 | CCCCCATCAGACCATCTACA | TTCGAGTCATGGCTCCTGTT |
| Klb | GATGAAGAATTTCCTAAACCAGGTT | AACCAAACACGCGGATTTC |
| Fgfr1b | CAACTTGCCGTATGTCCAGATC | CTCCGCATCCGAGCTATTAA |
| Fgfr1c | GCCAGACAACTTGCCGTATG | ATTTCCTTGTCGGTGGTATTAACTC |
| Fgfr4 | CGCCAGCCTGTCACTATACAAA | CCAGAGGACCTCGACTCCAA |
| Fbxo32 | TGTTACCCAAGGAAAGAGCAGTATGGA | ACGGAGCAGCTCTCTGGGTTATTG |
| Ttrim63 | TGGGGGAGCCACCTTCCTCT | ATGTTCTCAAAGCCCTGCTCTGTCT |
| FoxO1 | CCCAGGCCGGAGTTTAACC | GTTGCTCATAAAGTCGGTGCT |
| AChRα | AAGCTACTGTGAGATCATCGTCAC | TGACGAAGTGGTAGGTGATGTCCA |
| AChRβ | ACGGTCCACAACCATGGC | CATCATCGCTCACCCCAC |
| AChRγ | CTTGTGGCTAAGAAGGTGCCTG | GCAAGGACACATTGAGCACGAC |
| AChRδ | GAATGAGGAACAAAGGCTGATCC | GGTGAGACTTAGGGCGACAT |
| AChRε | AGACCTGAGGACACTGTCACCA | TCGTCCTTGCTGTAGTTGAGCC |
| Lrp4 | GCTATGGCAGAGCCTAGAGAA | CGACCAGCGTAGTCGATGG |
| Musk | TACAGAGGGGAGGTGTGTGAT | TCCCGGTAGGAGGTGTTGAA |
| App | TCCGAGAGGTGTGCTCTGAA | CCACATCCGCCGTAAAAGAATG |
| Dok7 | ATGCTGGTCTACAAGGACAAATG | AGCTACTGTCACATGGAACCT |
| Hdac4 | CTGCAAGTGGCCCCTACAG | CTGCTCATGTTGACGCTGGA |
| Tgfb1 | CTCCCGTGGCTTCTAGTGC | GCCTTAGTTTGGACAGGATCTG |
| Pai1 | TTCAGCCCTTGCTTGCCTC | ACACTTTTACTCCGAAGTCGGT |
| Ctgf | GGGCCTCTTCTGCGATTTC | ATCCAGGCAAGTGCATTGGTA |
| Smad3 | CACGCAGAACGTGAACACC | GGCAGTAGATAACGTGAGGGA |
| c-Jun | CCTTCTACGACGATGCCCTC | GGTTCAAGGTCATGCTCTGTTT |
| c-Fos | CGGGTTTCAACGCCGACTA | TTGGCACTAGAGACGGACAGA |
| Pdgfra | TCCATGCTAGACTCAGAAGTCA | TCCCGGTGGACACAATTTTTC |
| F4/80 | TGACTCACCTTGTGGTCCTAA | CTTCCCAGAATCCAGTCTTTCC |
| Itga7 | CTGCTGTGGAAGCTGGGATTC | CTCCTCCTTGAACTGCTGTCG |
| Cd31 | CTGCCAGTCCGAAAATGGAAC | CTTCATCCACCGGGGCTATC |

**Table S3. Post hoc power analyses.**
